# Unraveling dynamics of paramyxovirus-receptor interactions using nanoparticles displaying hemagglutinin-neuraminidase

**DOI:** 10.1371/journal.ppat.1012371

**Published:** 2024-07-25

**Authors:** Xuesheng Wu, Maite Goebbels, Oliver Debski-Antoniak, Katherine Marougka, Lemeng Chao, Tony Smits, Tom Wennekes, Frank J. M. van Kuppeveld, Erik de Vries, Cornelis A. M. de Haan

**Affiliations:** 1 Section Virology, Division Infectious Diseases and Immunology, Department Biomolecular Health Sciences, Faculty Veterinary Medicine, Utrecht University, Utrecht, the Netherlands; 2 Department Chemical Biology and Drug Discovery, Utrecht Institute for Pharmaceutical Sciences and Bijvoet Center for Biomolecular Research, Utrecht University, Utrecht, The Netherlands; Williams College, UNITED STATES OF AMERICA

## Abstract

Sialoglycan-binding enveloped viruses often possess receptor-destroying activity to avoid being immobilized by non-functional decoy receptors. Sialic acid (Sia)-binding paramyxoviruses contain a hemagglutinin-neuraminidase (HN) protein that possesses both Sia-binding and -cleavage activities. The multivalent, dynamic receptor interactions of paramyxovirus particles provide virion motility and are a key determinant of host tropism. However, such multivalent interactions have not been exhaustively analyzed, because such studies are complicated by the low affinity of the individual interactions and the requirement of high titer virus stocks. Moreover, the dynamics of multivalent particle-receptor interactions are difficult to predict from Michaelis-Menten enzyme kinetics. Therefore, we here developed Ni-NTA nanoparticles that multivalently display recombinant soluble HN tetramers via their His tags (HN-NPs). Applying this HN-NP platform to Newcastle disease virus (NDV), we investigated using biolayer interferometry (BLI) the role of important HN residues in receptor-interactions and analyzed long-range effects between the catalytic site and the second Sia binding site (2SBS). The HN-NP system was also applicable to other paramyxoviruses. Comparative analysis of HN-NPs revealed and confirmed differences in dynamic receptor-interactions between type 1 human and murine parainfluenza viruses as well as of lab-adapted and clinical isolates of human parainfluenza virus type 3, which are likely to contribute to differences in tropism of these viruses. We propose this novel platform to be applicable to elucidate the dynamics of multivalent-receptor interactions important for host tropism and pathogenesis, particularly for difficult to grow sialoglycan-binding (paramyxo)viruses.

## Author summary

Multivalent dynamic interactions of sialoglycan-binding viruses, including several paramyxoviruses, are a key determinant of host tropism and pathogenesis as they allow virion motility and escape from decoy receptors to find the functional receptors at the cell surface. To facilitate analysis of these interactions by biolayer interferometry, we developed system nanoparticles that multivalently present paramyxovirus hemagglutinin-neuraminidase (HN) proteins (referred to HN-NPs), thereby omitting the need of growing high titer virus stocks. Using the HN-NP system, we performed a detailed analysis of the importance of residues in the catalytic site and the second sialic acid-binding site (2SBS) of Newcastle disease virus (NDV) for multivalent dynamic receptor interactions. Our results show that the observed phenotypes are difficult to predict from Michaelis-Menten enzyme kinetics. In addition, we analyzed long-range effects between the two sites. Finally, we applied this system to compare the dynamic receptor-interactions of various parainfluenza viruses, collectively resulting in novel insights into the dynamics of multivalent-receptor interactions of paramyxoviruses.

## Introduction

Many respiratory viruses exploit sialoglycans as receptors for cell entry, including orthomyxoviruses, coronaviruses and paramyxoviruses [1,2]. Sialoglycan-binding viruses must prevent immobilization by non-functional decoy receptors abundantly present in the heavily sialylated respiratory mucus and on the cell surface [3,4]. Enveloped viruses often employ sialoglycan receptor-cleaving enzymes for the release of (newly assembled) virions from decoy receptors. These enzymes include neuraminidase proteins of influenza A and B viruses, hemagglutinin-esterase (HE) of embecoviruses, and hemagglutinin-neuraminidase (HN) proteins of paramyxoviruses [1,2]. Sialoglycan-binding and -cleavage properties of these viruses are balanced to the sialoglycan repertoire of the host and important for host tropism, and has been studied for IAV [5–8], coronavirus [9] and some paramyxoviruses [10–14].

Sialoglycan-binding virus particles typically engage a receptor surface by multiple low-affinity interactions with individual sialic acids (Sia). This results in a dynamic multivalent interaction of high avidity which, in the absence of receptor-destroying activity, will immobilize a virion on a receptor surface. Virion motility is essential to infection and relies on low-affinity receptor interactions combined with receptor-destroying activity [8,15–17]. For example, influenza A viruses (IAVs) bind multiple receptors with low-affinity (K_D_ ~1–20 mM) via their hemagglutinin (HA) trimers [18]. In the absence of NA activity, the high avidity of these interactions immobilizes the virions and prevents dissociation [19,20]. When NA is active, its sialidase activity reduces the sialoglycan density, resulting in movement of virions to nearby spots with higher sialoglycan density [16]. This mechanism of NA-driven directional virion motility has been referred to as a burnt-bridge ratchet (i.e., it cannot return on its track) or lawnmower molecular motor [21,22].

Unlike IAVs, paramyxoviruses combine receptor-binding and -cleaving activities within a single HN protein [23,24] displaying receptor-binding and -cleavage in the same primary Sia-binding site (Site I). HN is a type II membrane protein containing an N-terminal transmembrane domain (TM), a stalk region and a C-terminal globular head domain [23,24]. HN is presumably presented at the viral surface as a tetramer (or dimer of dimers) for most paramyxoviruses, including NDV [25,26] and PIV5 [23,25,27]. Chemical cross-linking studies also suggested a tetrameric hPIV3 HN [28], whereas recent structural analysis indicated a dimeric structure on the intact virus surface [29,30]. Analyses of the dissociation constant (K_D_) and enzymatic Michaelis-Menten constant (K_m_) indicate that HN is a neuraminidase that can hold its substrate long enough to function as a receptor-binding protein [31,32]. In addition, some HN proteins were shown to contain a 2^nd^ Sia binding site (2SBS or Site II), which for NDV is located at the dimer interface [33,34]. Removal of an N-glycan from hPIV1 [35] or hPIV3 [36] enabled virus binding in the presence of site I-inhibitors, presumably by unblocking a 2SBS, while a functional 2SBS in hPIV3 was also reported to be restored by another substitution (H552Q) [37,38]. For SeV, binding and cleavage are, proposedly, associated with two sites [39,40], but structural evidence for a 2SBS is lacking.

Except for receptor-interaction (binding and cleavage), HN also functions to stabilize the fusion (F) protein in a prefusion state, before engaging a Sia receptor [41]. After receptor binding, HN also activates F, resulting in conformational changes in F that drive virus-cell fusion [12,29,34,42–45]. Balancing the four activities of HN (F stabilization, receptor binding, F activation and receptor cleavage) is crucial for viral infection, pathogenesis and viral fitness [12–14,29,30,45–50] and is likely adapted to the host species-specific sialoglycome. In line herewith, the functional balance between HN receptor binding and cleavage, and fusion activation is significantly different between laboratory-adapted and clinical isolates [14,46,49].

Typically, HN-containing paramyxoviruses exhibit a preference for terminally-located Sias attached by a NeuAcα2-3Gal linkage (α2–3 Sia) [2,33,51–57] in contrast to human IAVs that prefer a NeuAcα2-6Gal linkage type (α2–6 Sia) [58]. Assays employing biolayer interferometry (BLI) [20,59–61] and fluorescence microscopy [8,17,62,63] enable analysis of the kinetics of virus-receptor interactions. Using BLI, it was shown that paramyxoviruses display sialidase-driven virion motility on receptor-coated surfaces, which was shown to differ for different paramyxoviruses [60]. BLI requires virus stocks with high virus particle numbers, the production of which in immortalized cells may introduce cell culture adaptations. For instance, clinical isolates of human parainfluenza 3 (hPIV3) poorly grow in immortalized cells resulting in the selection of cell culture-adaptive mutations [49]. Furthermore, substitution of functionally important residues may complicate the generation of viruses by reverse genetics and/or impact viral growth [64] thus preventing detailed BLI analyses. Finally, biosafety issues and gain-of-function restrictions may profoundly limit investigations on virus-receptor interactions.

Virus-receptor interaction studies using soluble recombinant glycoproteins have been successfully employed for IAV HA and NA [65,66]. Their artificial arrangement does however inadequately address the critical importance of multivalency and high avidity of genuine virus-receptor interactions, potentially leading to a biased focus on high-affinity receptors. Presentation of viral glycoproteins on beads, to better mimic virions, increases avidity and may enable binding to low-affinity receptors. Display of viral glycoproteins on self-assembling protein nanoparticles has already been exploited to induce increased activation of B cell receptors [67–69]. These nanoparticles do not mimic, however, virions in respect to size (lumazine synthase ~15nm [68], mi3 ~26nm [67], ferritin ~10nm [69]) and reduced viral glycoprotein density.

In this study, we employed virion-sized abiotic Ni-NTA nanoparticles, to which we conjugated tetrameric his-tagged HN glycoproteins of different paramyxoviruses. The resulting HN-NPs displayed similar receptor-interaction dynamics as paramyxoviruses including virion-like motility on a receptor-coated surface. Application to NDV confirmed and identified residues important for receptor binding and/or cleavage and allowed us to study the long-range effects between the catalytic site and the 2SBS. The system also enabled a comparative analysis of receptor binding dynamics of human and murine PIV1 and lab-adapted and clinical isolates of hPIV3.

## Results

### Synthesis of recombinant NDV HN proteins and coupling to Ni-NTA nanoparticles

**Fig 1:**
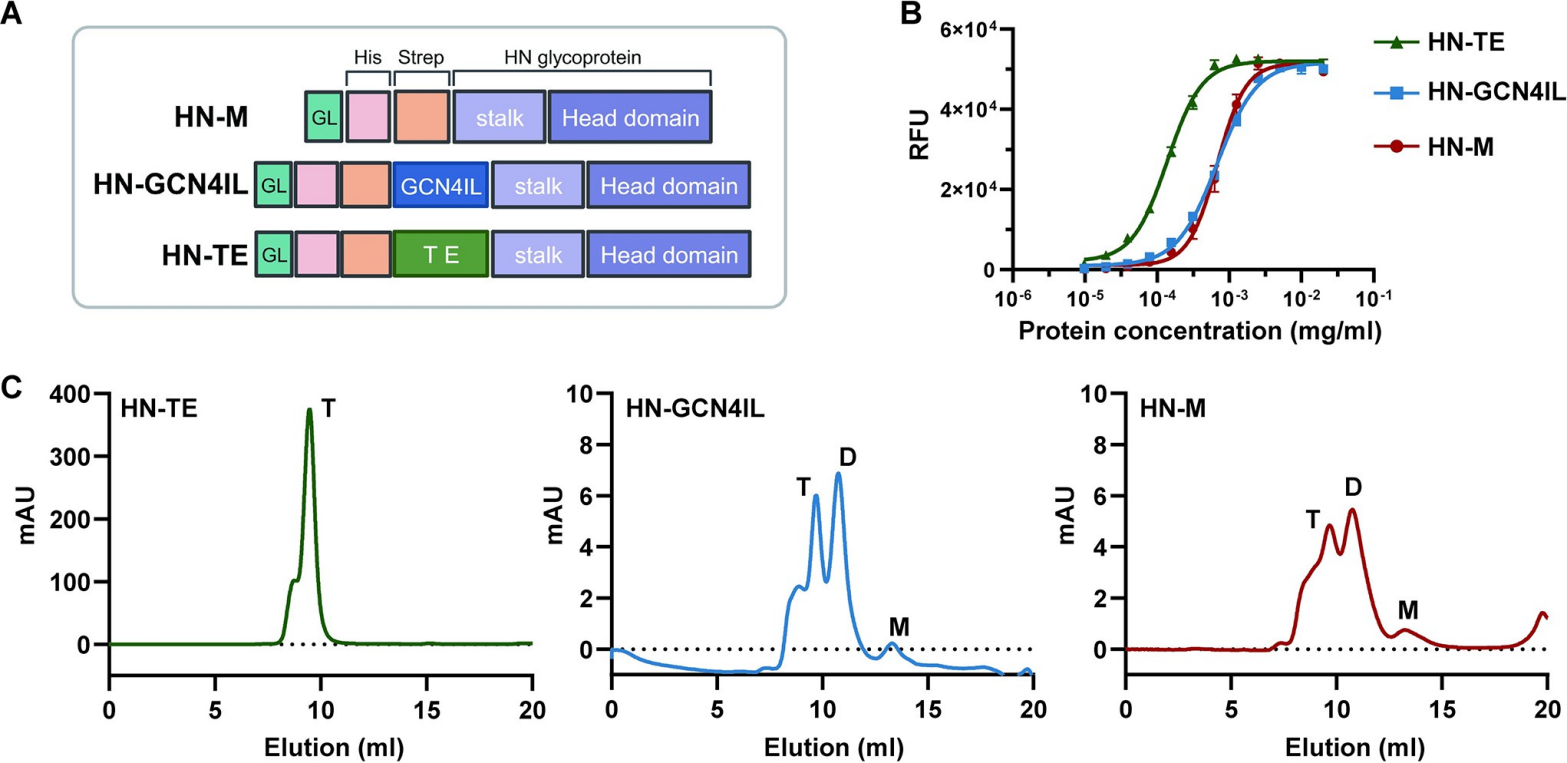
Expression of recombinant soluble NDV HN glycoproteins. (A) Schematic representation of recombinant HN-M (no oligomerization domain), HN-GCN4 IL (containing a GCN4 IL dimerization between the Strep-tag and the stalk) and HN-TE (containing a TE tetramerization domain between the Strep-tag and the stalk). His refers to the 6His tag, and GL refers to the signal peptide sequence of Gaussia luciferase. (B) Sialidase activity of serial two-fold dilutions of HN-TE, HN-GCN4 IL and HN-M was assessed at 37°C using the fluorogenic substrate 4-MUNANA at pH 7.0. Means in relative fluorescent units (RFU) and standard deviations are graphed. A representative experiment (n = 3) is shown. (C) Profile of size-exclusion chromatography (SEC) analysis on a Superdex 200 Increase 10/300 GL column of freshly purified NDV HN proteins. T, tetramer; D, dimer; M, monomer. Fig 1A created with Biorender.com.

To facilitate the analysis of multivalent, dynamic receptor interactions of sialoglycan-binding paramyxoviruses, which requires high titer virus stocks, we set out to develop nanoparticles to which soluble oligomeric HN proteins are coupled. As it has been reported that soluble HN proteins may adopt different oligomerization states, depending among others on the presence or absence of a stalk domain [25,27,70], we first designed different constructs to express the NDV HN ectodomain, including the stalk domain, with and without (latter referred to as HN-M) oligomerization domains (Fig 1A). The oligomerization domains included the GCN4IL dimerization [71] and tetrabrachion tetramerization domain [72] (referred to as HN-GCN4IL and HN-TE, respectively). All constructs were expressed in HEK293F cells and purified from cell culture supernatants (Fig 1B). Size-exclusion chromatography analysis (SEC) displayed variable amounts in monomeric, dimeric, and tetrameric forms for the different constructs. HN without an oligomerization domain or with the GCN4IL dimerization domain were both present as dimers and tetramers (Fig 1C). Incorporation of the tetrabrachion oligomerization domain led to a uniform tetrameric conformation (Fig 1C), which displayed the highest sialidase specific activity (Fig 1B). These results and the reported tetrameric conformation of NDV HN, let us to select HN-TE for subsequent analysis of receptor-interactions.

**Fig 2:**
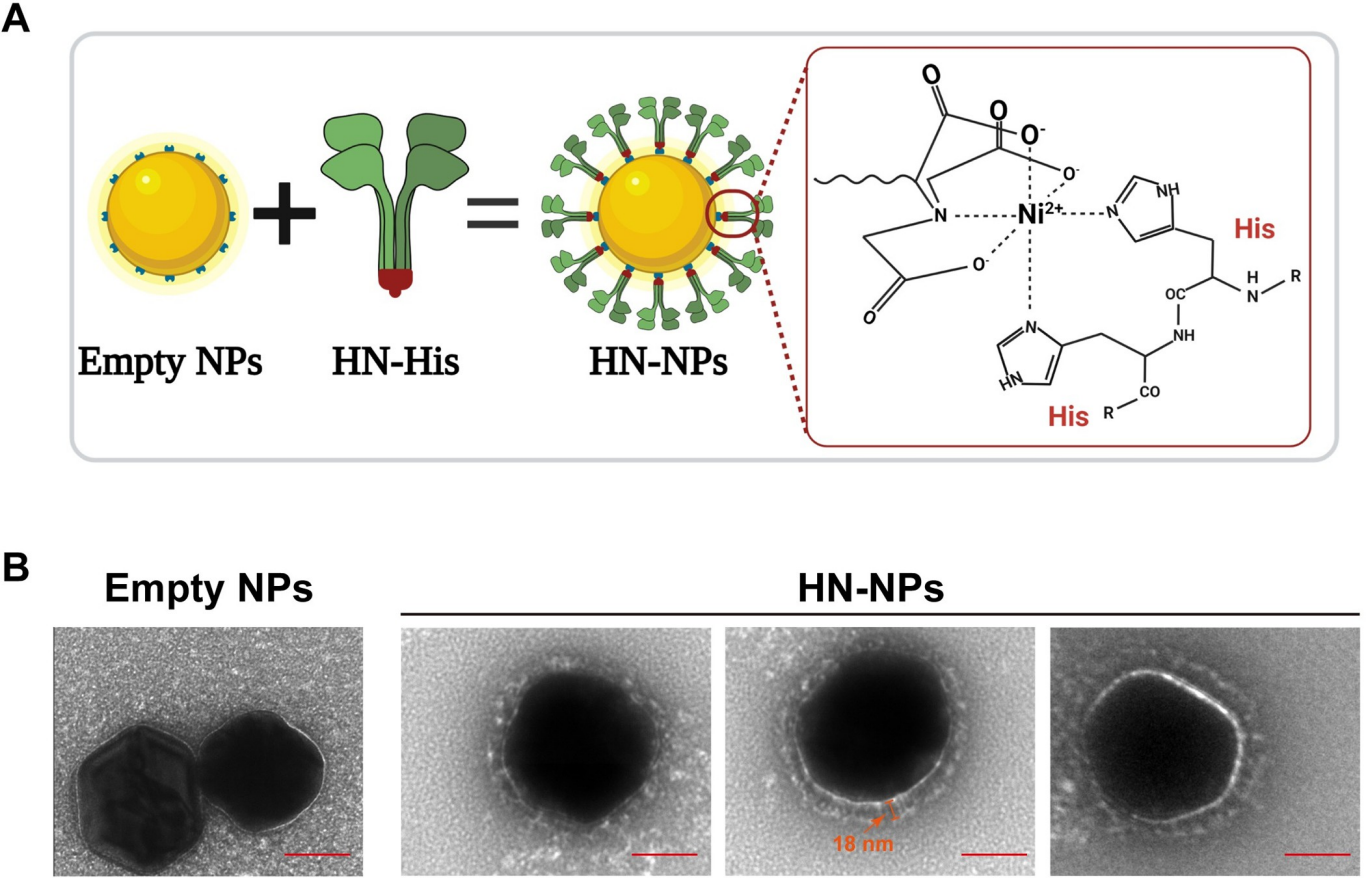
Characterization of HN-containing gold nanoparticles by negative stain TEM. (A) Schematic diagram illustrating the coupling of HN to 100 nm Ni-NTA gold nanoparticles via the N-terminal 6His tag. (B) Negative stain image of empty 100 nm Ni-NTA gold nanoparticles (left) or coupled with NDV HN (HN-NPs) (right). HN tetramers extended 18 nm from the nanoparticle surface. Scale bar, 50 nm. Fig 2A created with Biorender.com.

To assemble HN-containing nanoparticles (HN-NPs), we introduced a 6xhistidine (6His)-tag at the N-terminal end of the soluble HN proteins (Fig 1A), enabling spontaneous assembly of the HNs with nickel (II) nitrilotriacetic acid complex (Ni-NTA)-coated nanoparticles, thereby forming HN-NPs (Fig 2A). These HN-NPs display properly-oriented HN proteins, similar to virions [73] as shown by electron microscopical analysis (Fig 2B).

### Dynamics of HN-NP-receptor interactions

**Fig 3:**
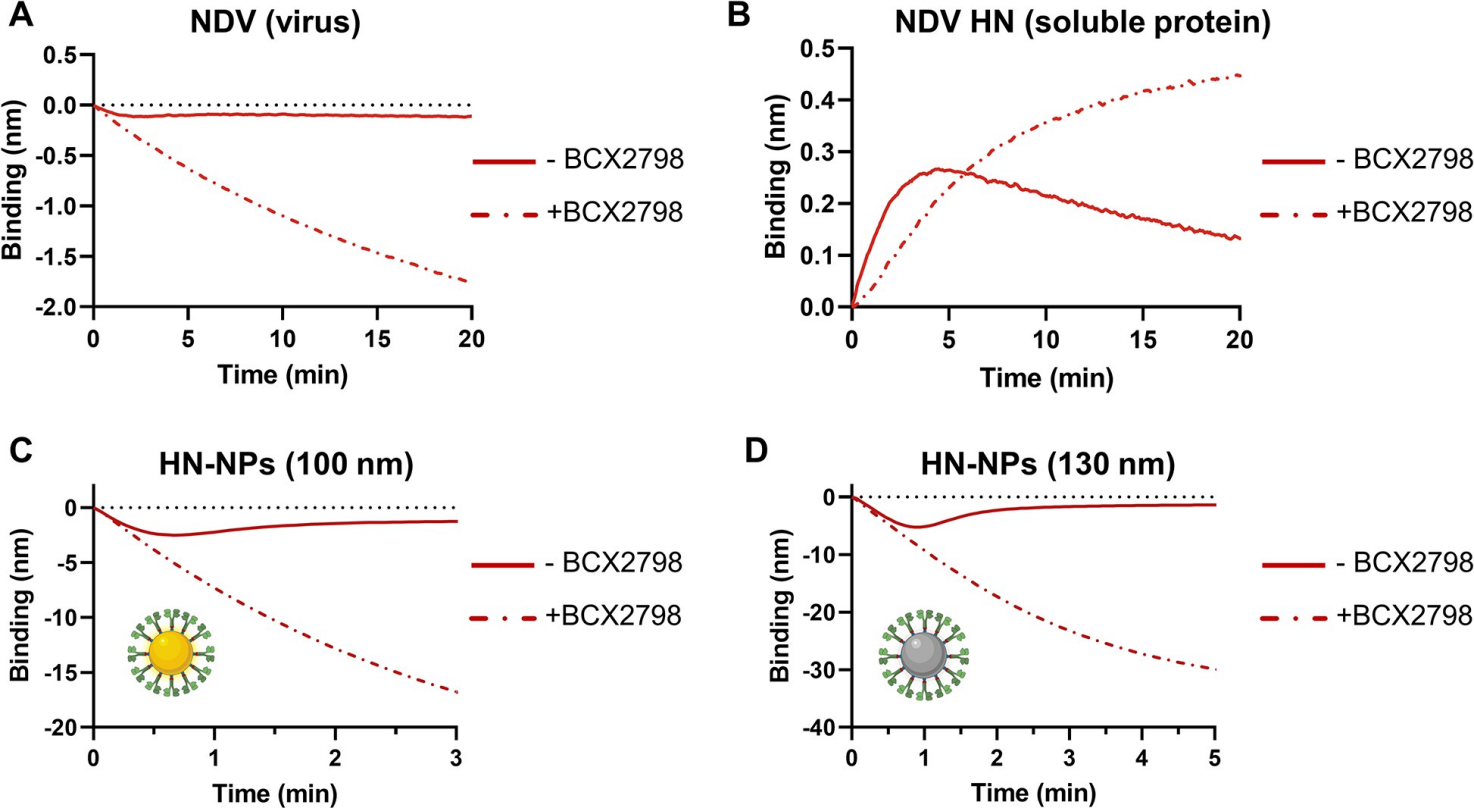
Kinetic analysis of virion-, HN- and HN-NP-receptor interactions using BLI. Streptavidin sensors were loaded to saturation with 3’S(LN)_3_. Subsequently, the sensor was incubated with **(A)** 1.0 x 10^9^ NDV virions, **(B)** 1 μg NDV HN, **(C)** 0.45 μg HN coupled to 4.41 x 10^8^ gold nanoparticles, **(D)** 0.45 μg HN coupled to 7.43 x 10^8^ Ni-NTA nanoparticles in the absence or presence of catalytic site inhibitor BCX2798. Particle numbers indicated are according to NTA analysis (also see S1 Table). Fig 3C and 3D created with Biorender.com.

Multivalent virus-receptor interactions display complex dynamics that can be kinetically monitored in real-time using BLI [20,60,74]. BLI is an optical technique for measuring macromolecular interactions by analyzing interference patterns of white light reflected from the surface of a biosensor tip. We studied to what extent HN-NPs display similar dynamics of virion-receptor interactions as virions. To this end, interactions of NDV and the nanoparticles with a 3’S(LN)_3_ receptor-coated surface were studied in the absence or presence of Sia analog BCX2798 (4-azido-5-isobutyrylamino-2,3-didehydro-2,3,4,5-tetradeoxy-d-glycero-d-galacto-2-nonulopy-ranosic acid), which binds and blocks the catalytic site (site I), but not the 2SBS (site II) of NDV [33,60,75,76]. In the absence of the inhibitor, NDV displayed a low negative binding signal, which after 1–2 minutes decreased again (became less negative), indicative of HN-NPs dissociation exceeding association (Fig 3A). The negative binding signal observed in BLI upon binding of virions is poorly understood but correlates with the size of these particles. While binding of smaller compounds like synthetic glycans or soluble proteins to the sensor surface results in a positive binding signal, binding of enveloped virions and vesicles consistently results in a negative binding curve [60,74,77]. Please note that in several previous studies the negative binding signal observed for virions was converted into a positive binding signal [20,61,78]. In the presence of BCX2798, binding of NDV to 3’S(LN)_3_ was increased (more negative) and no dissociation of NDV was observed (Fig 3A). These results are similar to those reported previously [60] and are in agreement with the presence of a 2SBS in NDV HN. Next, we studied the receptor interactions of unconjugated HN and HN-NPs (Fig 3B–3D). Also for soluble HN tetramers binding (positive signal) was followed by dissociation that could be blocked by BCX2798. Upon conjugation of HN protein to 100 nm gold nanoparticles similar binding curves in BLI analysis were observed as for NDV virions carrying the same HN (Fig 3C). The higher negative binding signal is likely due to increased light reflection of nanoparticles in comparison to virions. Minor binding was observed when HN was coupled to smaller (30–80 nm) gold nanoparticles (S1 Fig). 130 nm dextran iron oxide composite Ni-NTA nanoparticles displayed similar binding kinetics as the 100 nm gold nanoparticles and the NDV virions (Fig 3D), while binding was diminished for 250 nm dextran iron oxide composite nanoparticles (S1 Fig). Similar results were obtained when Zanamivir rather than BCX2798, albeit at sufficiently high concentration (S2 Fig), in agreement with a previous study [33]. All nanoparticle preparations bound similar amounts of HN as determined by western blot analysis (S1 Fig). Increasing the amount of HN coupled to the 130 nm dextran nanoparticles, above the amount used in Fig 3, did not increase the binding signal in BLI, while the signal decreased with lower amounts of HN (S3 Fig). We conclude that 100–130 nm nanoparticles coupled with HN protein display similar kinetics of virus binding as NDV virions. The reduced binding of smaller and larger nanoparticles might be attributed to their size per se, potential differences in particle numbers (S1 Fig) and/or HN density on the particle surface (S1 Table). In view of the strong binding observed for the HN-coupled 130 nm dextran iron oxide composite nanoparticles (Fig 3D), their similar size to virions (~100 to 250 nm) [73] and them being cheaper than the gold particles, we further used the 130 nm dextran iron oxide composite nanoparticles. Unfortunately, severe aggregation of these nanoparticles, regardless of the absence or presence of HN, during the TEM procedure prevented microscopical analysis. However, only minor aggregation of the nanoparticles was observed by NTA (S4 Fig). In subsequent BLI experiments 0.45 μg HN coupled to 7.43 x 10^8^ 130 nm nanoparticles was used except when indicated otherwise. Of note, the NDV HN-NPs displayed binding to sensors containing 3’S(LN)_3_, but not 6’S(LN)_3_ (S5 Fig) similarly as observed for NDV carrying the same HN protein [60], while empty nanoparticles did not bind to any of the synthetic glycans (S6 Fig).

### Motility of HN-NPs on a receptor coated surface

**Fig 4:**
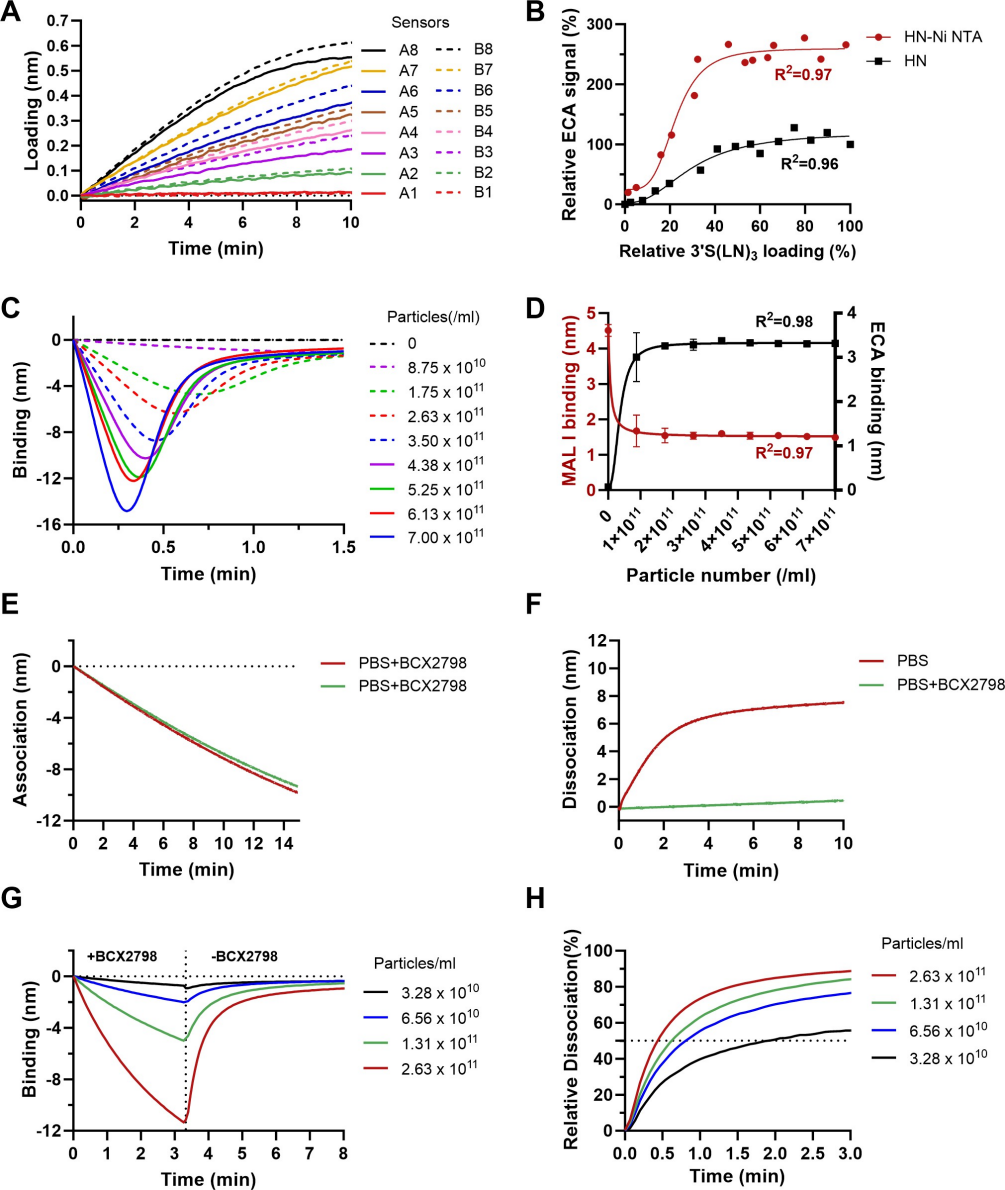
Sialidase-driven mobility of NDV HN-NPs on a receptor-coated surface. (A) Streptavidin sensors were loaded with varying densities of 3’S(LN)_3_ receptors. (B) Subsequently, these sensors were incubated with NDV HN-NPs or the corresponding amount unconjugated HN (0.45 ug) for 30 s. Cleavage of 3’S(LN)_3_ by HN-NPs and soluble HNs was probed using ECA lectin, which binds the Galβ1-4GlcNAc glycotope present after desialylation. The ECA signals (relative to the maximum signal obtained with soluble HN) were plotted against the relative receptor density. Results from three independent experiments combined are shown. (C) A diverse range of HN-NP concentrations was allowed to interact with 3’S(LN)_3_ loaded sensors for 10 min, followed by sensor regeneration in 10 mM Tris/Glycine buffer (pH 2.0). (D) The presence of remaining sialoglycan was probed by analyzing binding with MAL I (left Y-axes; red line) or ECA (right Y-axes; black line). MAL I specifically binds to the Neu5Acα2-3Galβ1-4GlcNAc glycotope, while ECA binds to the non-sialylated form thereof. (E) Binding of NDV HN-NPs (standard amount) to 3’S(LN)_3_ loaded sensors in the presence of BCX2798. (F) Subsequently, the sensors were moved to PBS, with or without BCX2798, to monitor sialidase driven self-elution of HN-NPs. (G) Binding of different concentrations of NDV HN-NPs to 3’S(LN)_3_ loaded sensors in the presence of BCX2798, followed by dissociation of HN-NPs in PBS. (H) Self-elution shown in (G) graphed relative to the binding level prior to self-elution. HN-NP concentrations indicated are to manufacturer’s specifications (2.63 x10^11^/ml corresponds to the standard amount).

To investigate the dynamic receptor interactions of the multivalent HN-NPs in more detail, we first analyzed the ability of unconjugated and nanoparticle-conjugated HN proteins to cleave Sia from a receptor-coated surface. To this end, sensors were loaded with varying densities of 3’S(LN)_3_ in duplicate (Fig 4A) and exposed to either HN-NPs or the same amount of soluble HNs (assuming 100% coupling efficiency) for 30 seconds. Next, cleavage of sialoglycan receptors by HN was analyzed using the ECA lectin, which selectively recognizes epitopes containing terminal Galβ1-4GlcNAc. Notably, the HN-NPs cleaved two-fold more receptors across various receptor densities in comparison to soluble HN proteins, as evidenced by the higher ECA binding signal (Fig 4B). Of note, coupling of HN to NPs did not affect the sialidase activity as determined using the small molecule substrate 4-MUNANA (S7 Fig). The HN-NPs also displayed much higher hemagglutinating activity than a similar amount of unconjugated HN (S8 Fig).

Building on our previous methods for assessing NDV virion motility [60], we studied potential HN-NP motility. Sensors, saturated with 3’S(LN)_3_, were subjected to incubation with varying concentrations of NDV HN-NPs (Fig 4C). At higher particle numbers, peak height increased while the time to reach this peak was decreased, in agreement with previous results [60]. Next, we quantified the extent of receptor cleavage by analyzing MAL I and ECA lectin binding. Binding of MAL I to 3’S(LN)_3_ decreased, while ECA binding to (LN)_3_ increased upon incubation of the sensors with different concentrations of HN-NPs. Binding of these lectins, was however, independent of the NP concentration used. At approximately 15% of the maximum HN-NP concentration analyzed, the same modification on the sensor surface was observed (Fig 4D). These observations are consistent with prior findings with NDV virions [60]. To further validate the similarities between HN-NPs and virions, we monitored the release of HN-NPs from a receptor-coated surface in the absence or presence of BCX2798 (Fig 4F) after prior association of the HN-NPs to these sensors in the presence of BCX2798 (Fig 4E). As for virions [60], dissociation was fast in the absence of BCX2798, but essentially not observed in its presence (Fig 4F). Next, we analyzed binding of different amounts of NDV HN-NPs to 3’S(LN)_3_-coated sensors in the presence of BCX2798 (Fig 4G) and subsequently tracked their dissociation in the absence of BCX2798 (Fig 4H). In agreement with results obtained for NDV [60], relative dissociation of HN-NPs was faster with more nanoparticles associated to the sensor surface. In summary, these results show that HN-NPs modify a receptor-coated surface or hemagglutinate to a different extent than unconjugated HN. HN-NPs interact with a receptor-coated surface similarly as NDV virions, in agreement with a model in which HN-NPs and NDV virions move on and modify the receptor-coated surface until receptor density is reduced to a threshold resulting in particle release. This threshold is reached faster when more particles were associated to the sensor, in agreement with HN-NPs motility on such a surface. We conclude that HN-NPs provide an alternative approach for investigating NDV-receptor interactions.

### Functional analysis of NDV HN catalytic site mutations on dynamic receptor interactions of HN-NPs

**Fig 5:**
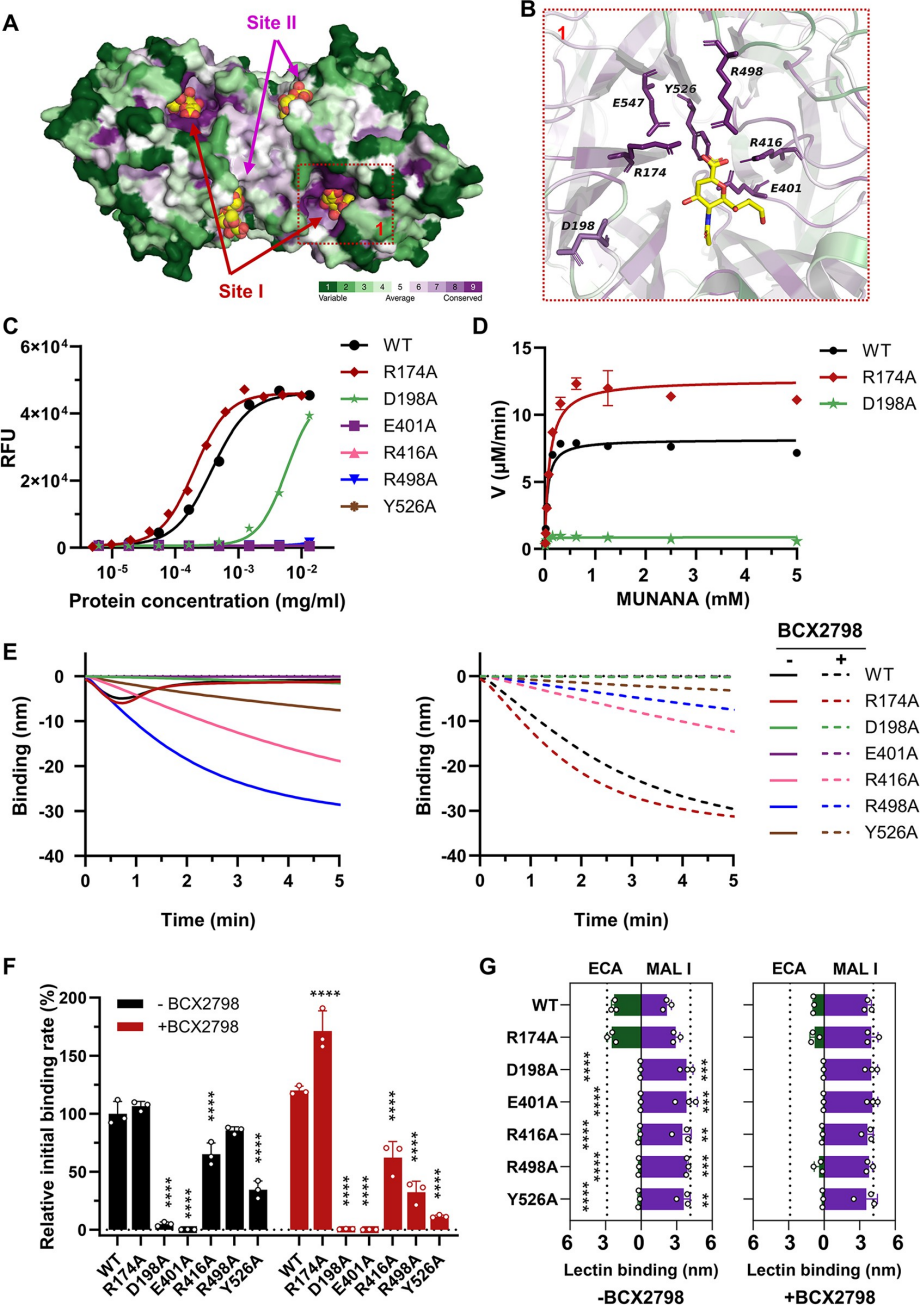
Unraveling the role of residues in the catalytic site (Site I) of NDV HN in dynamic receptor interactions using the HN-NP system. (A) Conservation analysis of the NDV HN glycoprotein (PDB ID:1USR) using the Consurf server color code, with green-through-purple corresponding to variable (grade 1) to conserved (grade 9) positions. Catalytic site (Site I) and 2SBS (Site II) occupied with Sia are indicated. (B) Magnified view of the catalytic site, with side chains shown for key residues involved in the interaction with Sia, which were selected for alanine substitution. (C) The sialidase activity of different amounts of NDV HN WT and mutant proteins was determined by applying the 4-MUNANA fluorometric assay under standard conditions. Specific activity (cleavage/amount of protein; 1/EC_50_) was determined from the linear parts of the curves and depicted normalized to HN WT in Table 1. (D) Enzyme kinetics analysis for NDV HN WT and mutants. Michaelis-Menten kinetics were determined for WT, R174A and D198A mutants. To this end, 0.2 μg of each protein was incubated with increasing concentrations of fluorogenic 4-MUNANA substrate (0.0024–5 mM). The reaction velocity (μM/min) of HNs is shown based on substrate conversion using a 4-methylumbelliferone (4-MU) standard curve. Mean values were determined from at least two independent experiments performed in triplicate and are presented with SDs indicated by error bars. From these graphs K_m_ and K_cat_ values shown in Table 1 were determined. (E) Streptavidin sensors were loaded to saturation with 3’S(LN)_3_ receptors. Subsequently, the sensors were incubated with standard amounts of NDV HN-NPs. HN-NPs binding curves were generated similarly as described in the Fig 4 legend using 3’S(LN)_3_ in the absence or presence of 1 mM BCX2798. (F) The initial binding rate of HN-NPs, corresponding to the steepness of the tangent at the beginning of the binding curves was determined from graphs as shown in E and normalized to the WT HN in the absence of BCX2798 (n  =  3, data are mean ± SD). Significance was analyzed using two-way ANOVA test compared to WT HN-NPs in the absence or presence of BCX2798. Significant differences between initial binding rates in the absence or presence of BCX2798 are shown in S3 Table. (G) Binding of MAL I and ECA after incubation of the sensors with the indicated HN-NPs and analyzed using two-way ANOVA test compared to the corresponding WT HN-NPs. MAL I binding in the absence of sialidase activity and ECA binding after treatment of the sensors with Arthrobacter ureafaciens NA (AUNA) are shown as dashed lines. Low binding levels of MAL I and high binding levels of ECA correspond with high levels of desialylation. * P≤0.05, ** P≤0.01, *** P≤0.001, **** P < 0.0001.

We used the nanoparticle system to study the effect of different catalytic site mutations, which are difficult to study in the context of virions, on the dynamics of receptor interactions. Based on the crystal structure of NDV HN (PDB: 1USR) [34] and previously published results (S2 Table; [44,79–82]), we introduced alanine substitutions within the catalytic site, including highly conserved residues R174, D198, E401, R416, R498, Y526 and E547 (Fig 5A and 5B). All but one (E547A) of these mutant HN proteins could be expressed and purified. First, we determined the specific activity of the resulting proteins using the small molecule substrate 4-MUNANA. Except for R174A, all substitutions severely impaired the relative specific activity (1/EC_50_) of HN (Fig 5C and Table 1). Next, we analyzed the Michaelis–Menten kinetics using 4-MUNANA within a range of 0.0024 to 5.0 mM for the D198A and R174A mutants, as well as the wild type (WT). The Michaelis-Menten constant (K_m_) represents the enzyme’s substrate affinity (Table 1). Although the D198A mutant displayed lower specific sialidase activity (Fig 5C), its affinity to the substrate was higher than that of WT and the R174A mutant (Table 1). Meanwhile, the reaction rate (K_cat_) of HN D198A, represented by the catalytic activity derived from V_max_ (Table 1) was much lower compared to WT and the R174A mutant. The catalytic efficiency (K_cat_/K_m_) of WT and R174A were similar and higher than that of D198A.

**Table 1:** Kinetic parameters of NDV HN proteins for cleavage of 4-MUNANA.

		K_cat_ (s^-1^)	K_m_ (μM)	K_cat_ / K_m_ (s^-1^M^-1^)	1/EC_50_^a^
	WT	4.75 ± 0.12	50.88 ± 6.21	93364.43 ± 9061.13	1.00 ± 0.00
Site I	R174A	7.34 ± 0.20	90.35 ± 11.39	81217.15 ± 7981.82	1.90 ± 0.46
	D198A	0.50 ± 0.02	14.03 ± 3.59	35758.20 ± 7725.55	0.07 ± 0.00
Site II	F156A	4.51 ± 0.25	511.60 ± 91.27	8814.44 ± 1093.10	0.08 ± 0.00
	S222A	14.49 ± 0.43	320.70 ± 32.66	45181.56 ± 3249.87	1.24 ± 0.03
	V517A	2.42 ± 0.14	437.10 ± 87.76	5541.46 ± 789.65	0.07 ± 0.00
	S519A	9.67 ± 0.29	118.10 ± 15.79	81892.08 ± 8457.06	3.14 ± 0.66
	L552A	6.09 ± 0.11	255.40 ± 18.10	23867.92 ± 1245.64	0.39 ± 0.01
	F553A	8.34 ± 0.22	585.10 ± 48.89	14259.66 ± 815.16	0.18 ± 0.02

^a^ Relative specific activity. Data normalized to WT.

To assess how these kinetic parameters relate to the dynamic receptor interactions of the nanoparticle, HN proteins were coupled to 130 nm Ni-NTA nanoparticles and subjected to BLI analysis in the presence or absence of BCX2798 inhibitor (Fig 5E). The interaction of the R174A mutant nanoparticles with the receptor-coated BLI sensor was similar to WT, with initial binding followed by release resulting from the sialidase activity. No release was observed in the presence of the catalytic site inhibitor BCX2798 (Fig 5E). Quantification of the initial binding rate (initial slope of the binding curve after starting receptor binding) [74] showed that WT and R174A HN-NPs have a similar initial binding rate in the absence of BCX2798. In the presence of BCX2798, the initial binding rate of R174A HN-NPs was 1.5-fold higher than that of WT (Fig 5F). Both HN-NPs modified the receptor-coated surface as determined by lectin binding (increased binding of ECA and decreased binding of MAL I) to the same extent, which was inhibited by BCX2798 (Fig 5G). No or very little binding was observed for E401A and D198A HN-NPs, respectively (Fig 5E and 5F). In the absence of appreciable binding, the D198A HN-NPs were not able to detectably cleave sialoglycans on the sensor surface (Fig 5G). Clear binding could be observed, however, for the sialidase-negative R498A, R416A and Y526A HN-NPs (Fig 5E), which did not decline over time in agreement with the absence of sialidase activity as determined in the 4-MUNANA assay (Fig 5C) and by lectin binding (Fig 5G). Binding of these HN-NPs could also be observed in the presence of BCX2798 (Fig 5E), albeit lower than observed for WT, indicative of these mutations in the catalytic site also affecting binding via the 2SBS to different extents. Binding of R498A and Y526A HN-NPs was negatively affected by the presence of BCX2798 (Fig 5F), indicating that in the absence of the inhibitor, receptor binding of HN-NPs occurs in part via the sialidase-negative catalytic site (Site I). In conclusion, the different catalytic site mutant proteins display different multivalent interaction dynamics with a receptor-coated surface in the context of HN-NPs, which do not necessarily correlate with the enzyme kinetics as determined in the 4-MUNANA assay. Our results indicate that catalytic site mutations may also affect binding in the presence of BCX2798, thus via the 2SBS and that a sialidase-negative catalytic site may contribute to particle binding.

### Functional analysis of NDV HN 2SBS mutations on dynamic receptor interactions of HN-NPs

**Fig 6:**
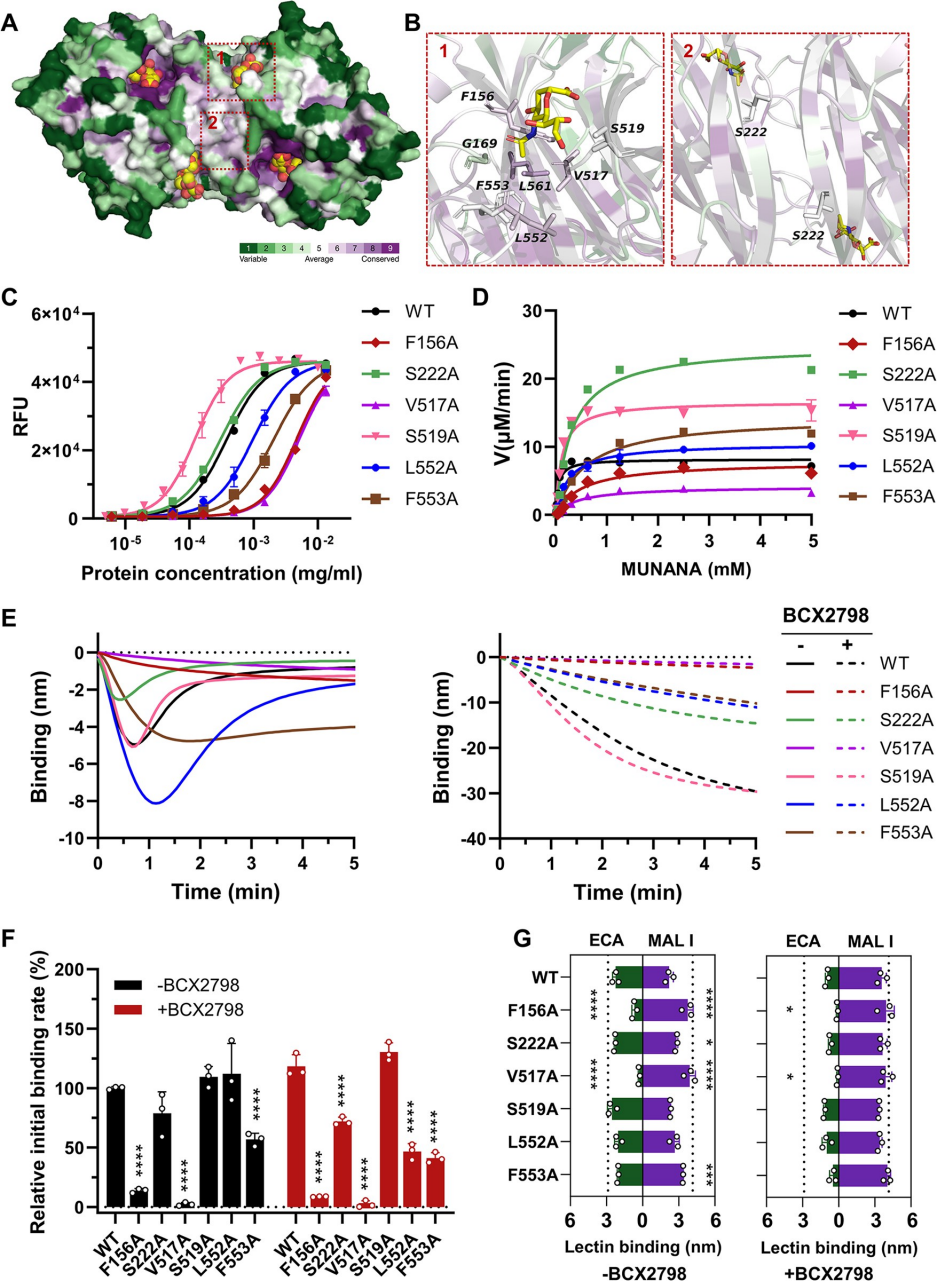
Unraveling the role of residues located in the dimer interface or 2SBS of NDV HN in dynamic receptor interactions using the HN-NP system. (A) Conservation analysis of the NDV HN glycoprotein dimer (PDB ID:1USR) using Consurf server color, with green-through-purple corresponding to variable (grade 1) to conserved (grade 9) positions. Catalytic site (Site I) and 2SBS (Site II) occupied with Sia are indicated. (B) Magnified views of residues selected for substitution. Left panel, residues located in the 2SBS with the side chains shown for key residues involved in the interaction with Sia. Right panel, membrane-proximal region of the interface with S222 indicated. (C) The sialidase activity of different amounts of NDV HN WT and mutant proteins was determined by applying the 4-MUNANA assay under standard conditions. Specific activity (cleavage/amount of protein; 1/EC_50_) was determined from the linear parts of the curves and depicted normalized to HN WT in Table 1. (D) Enzyme kinetics analysis for NDV HN WT and mutants was performed as described in the Fig 5 legend. K_m_ and K_cat_ values are shown in Table 1. Mean values were determined from at least two experiments performed in triplicate and are presented with SDs indicated by error bars. (E) HN-NPs binding curves were generated similarly as described in the Fig 5 legend using 3’S(LN)_3_ in the absence or presence of 1 mM BCX2798. (F) The initial binding rate of HN-NPs was determined from graphs as shown in E and normalized to the WT HN-NPs in the absence of BCX2798 (n  =  3, data are mean ± SD) and analyzed using two-way ANOVA test compared to the corresponding WT NPs. Significant differences between initial binding rates in the absence or presence of BCX2798 are shown in S3 Table. (G) Binding of MAL I and ECA after incubation of the sensors with the indicated NPs. Significance was analyzed using two-way ANOVA test compared to the corresponding WT NPs. MAL I binding in the absence of NA activity (no NA) and ECA binding after treatment of the sensors with AUNA are also shown in dash line. *, P≤0.05, **, P≤0.01, ***, P≤0.001, ****, P < 0.0001.

The dimer interface of NDV HN contains a 2SBS. To explore the role of the 2SBS in the dynamics of HN-NP interactions we conducted a mutational analysis targeting residues (F156, G169, V517, S519, L552, F553 and L561) located in the membrane-distal end, where the 2SBS is located. These residues have been reported to be important for the receptor binding activity of the 2SBS (S2 Table; [34,83]). Additionally, S222A, positioned in the membrane-proximal region of the interface, has previously been associated with compromised fusion activity [84] and impaired hemadsorption activity [85]. Compared to the highly conserved residues in Site I (Fig 5A and 5B), the conservation scores of these residues are lower (Fig 6A and 6B). Substitutions G169A and L561A disrupted the expression of HN in HEK293F cells, while other mutant HNs could be successfully expressed. To quantitatively assess the impact of the above-mentioned mutations, we first conducted sialidase kinetic assays using 4-MUNANA. All HNs, with the exception of S222A and S519A mutants, displayed reduced specific sialidase activity compared to WT HN (Fig 6C), with the K_cat_ of some being higher (S222A, S519A, L552A and F553A) or lower (V517A) than that of WT HN (Fig 6D and Table 1). All substitutions negatively affected the affinity of the HN proteins for 4-MUNANA, resulting in higher K_m_ values (Table 1). The catalytic efficiency of all mutant HN proteins was comparable (F156A, S419A) or lower (others) than that of WT HN. These results indicate that substitutions in the 2SBS may indirectly affect cleavage by the catalytic site, in agreement with a previous studies [83,86].

The multivalent dynamic receptor interactions of 2SBS-related mutant proteins were assessed using HN-NPs in the presence or absence of BCX2798 inhibitor (Fig 6E). The S519A HN-NPs behaved similar to WT HN-NPs, with or without BCX2798 inhibitor (Fig 6E–6G), indicating that substitution S519A did not negatively affect the 2SBS or the catalytic site (site I). While unconjugated S222A HN exhibited reduced affinity for 4-MUNANA (Fig 6D and Table 1), the initial binding rate of S222A HN-NPs was comparable to that of WT HN-NPs in the absence of the BCX2798 inhibitor. However, S222A HN-NPs displayed a faster release from the sensor surface. In the context of HN-NP system, F156A, V517A, L552A and F553A HN proteins showed distinct dynamic interactions. Both F156A and V517A HN-NPs bound to and cleaved the receptor-coated surface to a lesser extent than WT HN-NP. L552A HN-NPs showed similar initial binding rate to WT in the absence of BCX2798, while that of F553A HN-NPs mutants was lower. Release of both HN-NPs from the sensor surface was much slower than that of WT HN-NPs (Fig 6E and 6F), in agreement with their lower specific activity (Fig 6C). L552A and F553A HN-NPs displayed a similar initial binding rate in the presence of BCX2798, which was lower than that of WT HN-NPs and indicating that these substitutions negatively affect the 2SBS (Fig 6E–6G). An overview of all quantitative analyses of the NDV HN enzymatic assays is shown in S9 Fig. Together, these results indicate that substitutions in the 2SBS/dimer interface may not only affect the receptor binding properties of the 2SBS, but also the catalytic properties of site I, in agreement with previous studies [84,86], and as a result may alter the dynamic receptor-interactions of the HN-NPs in a way that is difficult to predict from Michaelis-Menten kinetics only.

### Investigation of dynamic receptor interactions of other parainfluenza viruses using the nanoparticle system

**Fig 7:**
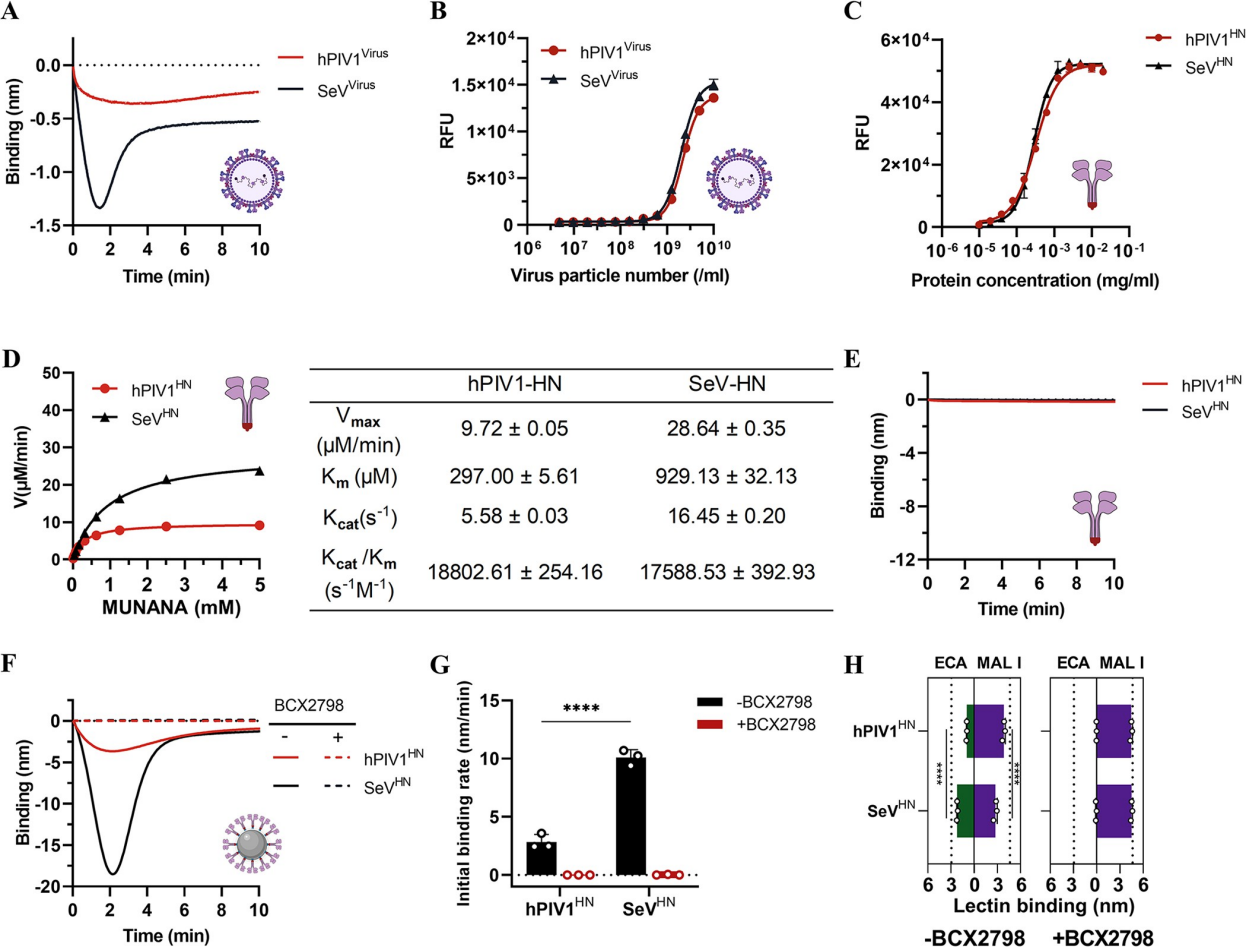
Applying the HN-NP system to compare dynamic receptor interactions of hPIV1 and SeV. (A) Virus binding curves were generated similarly as described in the legend to Fig 3 (1.0 x 10^9^ hPIV1 or SeV virions). (B) The sialidase activity of different concentrations of hPIV1 and SeV (starting concentration 1.0 x 10^10^ virus particles/ml) was assessed in triplicate using 4-MUNANA under standard conditions. Means and standard deviations are graphed. (C) The sialidase activity of different amounts of hPIV1 HN and SeV HN proteins were determined as described in the Fig 5 legend. (D) Enzyme kinetic analysis for hPIV1/SeV HN proteins was performed similarly as described in the Fig 5 legend. Enzyme kinetics parameters obtained from progress curve analysis for the 4-MUNANA substrate are shown in the table in the middle. (E) SA sensors loaded with saturate 3’S(LN)_3_ were reacted with 1 μg hPIV1/SeV-HN proteins for 10 mins to generate the binding curve. The binding of soluble hPIV1/SeV HN proteins was undetectable in BLI analysis. (F) Streptavidin sensors were loaded to saturation with 3’S(LN)_3_ receptors. Subsequently, the sensors were incubated with standard amounts of hPIV1/SeV HN-NPs. HN-NPs binding curves were generated similarly as described in the Fig 4 legend using 3’S(LN)_3_ in the absence or presence of 1 mM BCX2798. (G) The initial binding rate of HN-NPs was determined from graphs as shown in F (n  =  3, data are mean ± SD) and analyzed using one-way ANOVA test. (H) Binding of MAL I and ECA after incubation of the sensors with the indicated HN-NPs. Significance was analyzed using two-way ANOVA test. MAL I binding in the absence of sialidase activity and ECA binding after treatment of the sensors with AUNA are shown as dashed lines. **** P < 0.0001. Created with Biorender.com.

Sendai virus (SeV, murine parainfluenza virus 1) closely resembles its human counterpart human parainfluenza virus 1 (hPIV1), as evidenced by their high-sequence homology and antigenic cross-reactivity [87–89]. Both SeV and hPIV1 are known to bind α2-3-linked Sia [52,56,90], but whether they differ in their dynamics of virus-receptor interactions is unknown. Here we compared virions and HN-NPs to study these receptor interactions for hPIV1 and SeV using BLI. For both SeV and hPIV1, binding of virions to a receptor-coated sensor was followed by release, with the peak of SeV binding being higher than that of hPIV1 (Fig 7A). The dynamic receptor interactions of SeV virions was similar to what was observed previously [60]. Sialidase activity per virion was similar for the two viruses (Fig 7B), just as the specific activity of recombinantly expressed tetrameric HN proteins of hPIV1 and SeV was similar (Fig 7C). However, hPIV1 HN showed a higher substrate affinity than SeV, as evidenced by 3-fold lower K_m_ value, while its reaction rate K_cat_ was 3-fold lower (Fig 7D), resulting in a similar catalytic efficiency (Fig 7D). Unlike NDV HN, soluble HN from hPIV1 or SeV showed no binding to 3’S(LN)_3_ receptor in BLI analysis (Fig 7E). Coupling HNs to nanoparticles resulted in similar relative binding curves compared to virus, with SeV HN-NPs displaying a much larger binding peak and initial binding rate than hPIV1 (Fig 7A, 7F and 7G). Results demonstrated that neither hPIV1 nor SeV HN bound in the presence of BCX2798 (Fig 7F). While hPIV1 and SeV and their HN proteins exhibited similar sialidase activity using the 4-MUNANA assay (Fig 7B and 7C), SeV more extensively modified the receptor-coated surface, with higher ECA and lower MAL I binding compared to hPIV1 HN-NPs modified sensors (Fig 7H). In conclusion, hPIV1 and SeV display different kinetic receptor interactions which can be recapitulated using HN-NPs.

### Applying the HN-NP system to study the functional properties of hPIV3 HN from a laboratory-adapted strain (LS) and a clinic isolate (CI)

**Fig 8:**
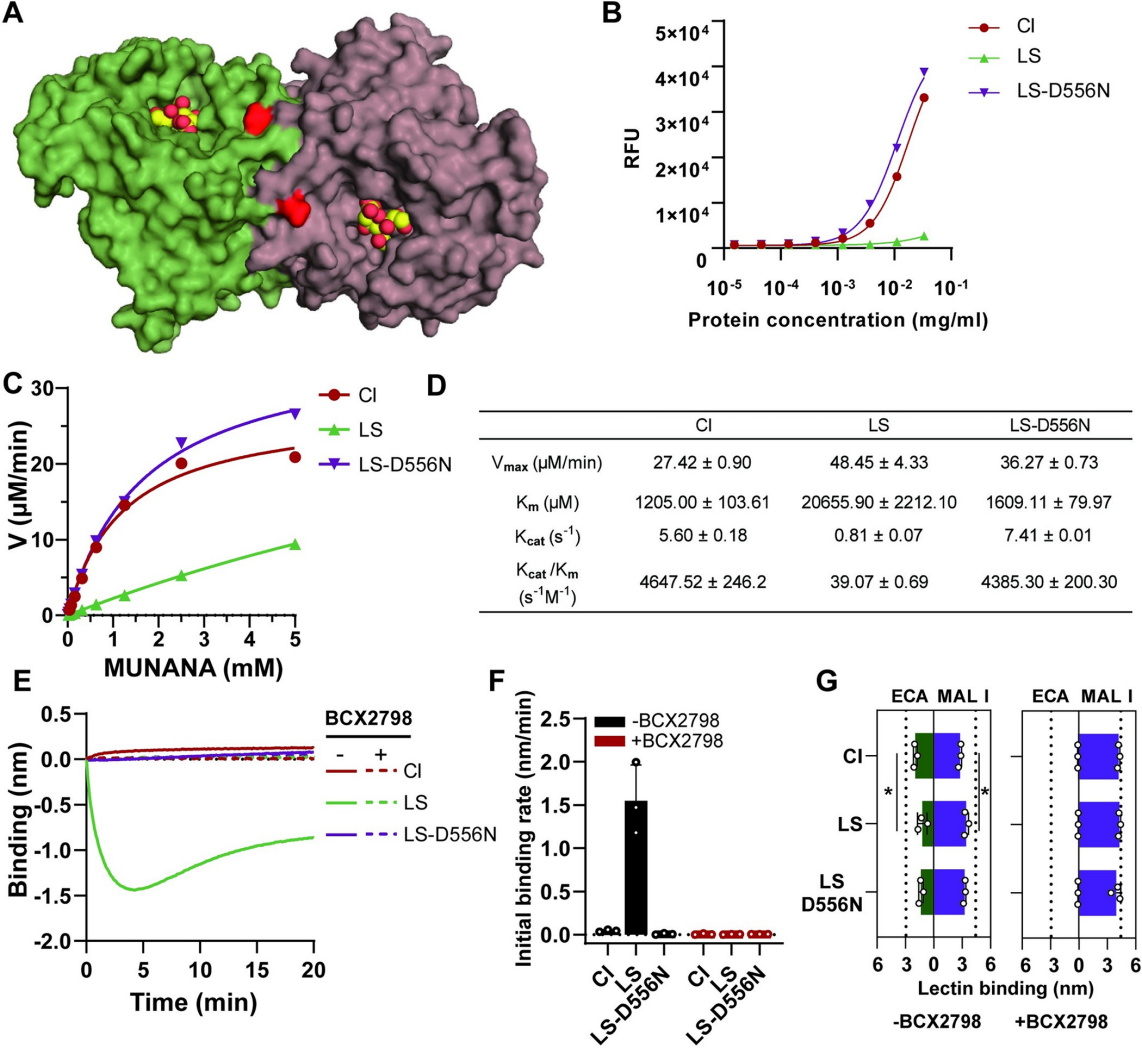
Applying the HN-NP system to compare the dynamic receptor interactions of a laboratory-adapted strain (LS) and a clinical isolate (CI) of hPIV3. (A) Structure of hPIV3 HN (PDB ID: 4MZA) homodimer is shown in a surface representation, with catalytic site occupied by Sia, D556 is shown in red. (B) Sialidase activity of serial two-fold dilutions of hPIV3 LS/CI HN proteins. (C) Enzyme kinetics analysis for hPIV3 LS/CI HN proteins was performed similarly as described in Fig 5 legend. (D) Kinetics parameters obtained from progress curve analysis for the 4-MUNANA substrate are shown in the table. (E) HN-NPs binding curves were generated similarly as described in the Fig 4 legend using 3’S(LN)_3_ in the absence or presence of 1mM BCX2798. (F) The initial binding rate of HN-NPs was determined from graphs as shown in E (n = 3, data shown are mean ± SD). (G) Binding of MAL I and ECA after incubation of the sensors with the indicated HN-NPs. Significance was analyzed using two-way ANOVA test. MAL I binding in the absence of NA activity (no NA) and ECA binding after treatment of the sensors with AUNA are shown as dashed lines. *, P≤0.05.

LS and CI of hPIV3 are known to differ in the enzymatic properties of their HN proteins, with HN of CI being more active than that of LS [46,48,49]. The residue D at position 556 (Fig 8A) has been reported to be key in this phenotypic difference and may be substituted by N within a single round of passage [14,49]. Based on a previous study, we selected a CI, which was directly sequenced without passaging in immortalized cells (S10 Fig) [49]. Here we analyzed the properties of HN-NPs decorated with HN of an LS (with N556) or this CI (with D556). The sialidase activity of soluble CI HN was markedly higher than that of LS HN, while substitution D556N in LS HN resulted in a similar sialidase activity as CI HN (Fig 8B). Substrate affinity of LS-D556N HN was also comparable to that of CI HN, which was approximately 20-fold higher than that of LS HN (Fig 8C and 8D). To test how the dynamic receptor-interactions of LS and CI differ, we employed the HN-NP system. While LS HN-NPs showed binding to the 3’S(LN)_3_ receptors with limited release, CI and LS-D556N HN-NPs did not (Fig 8E and 8F). Binding of LS HN-NPs was effectively inhibited by BCX2798 (Fig 8E and 8F). Despite the absence of detectable binding, CI HN-NPs modified the receptor-coated surface more than LS HN-NPs as determined by increased ECA and decreased MAL I binding, which was inhibited by BCX2798 (Fig 8G). These results demonstrate the applicability of HN-NPs to study multivalent dynamic receptor interactions for viruses that are prone to acquire cell culture adaptations.

## Discussion

Multivalent dynamic interactions between virions and sialoglycan receptors, which depend on the balance between receptor binding and cleavage, allow virion motility and are crucial for host tropism. These interactions can be studied in detail using BLI as was demonstrated recently for IAVs [20,59,61] and several paramyxoviruses [60], but are hampered by the need for high titer stocks. In this study, we developed a HN-NP system based on coupling of recombinant tetrameric HN ectodomains to nanoparticles and showed that the dynamic virus-receptor interactions of these HN-NPs are comparable with those of virions. Analyses of (mutant) HN proteins of different paramyxoviruses demonstrated the versatility of this system. Data presented here suggest that catalytic properties of HN proteins are affected by their multivalent display on nanoparticles making multivalent dynamic receptor interactions difficult to predict from Michaelis-Menten kinetics determined with small molecule substrates. This novel system will allow for a more detailed analysis of the dynamics of paramyxovirus-receptor interactions, beyond receptor-specificity, for example to elucidate to what extent these differences contribute to the host tropism of viruses.

100–130 nm tetrameric HN nanoparticles displayed similar receptor interaction dynamics as their counterpart NDV, hPIV1, hPIV3 and SeV virions. The NDV HN ectodomains formed dimers and tetramers in the absence of an oligomerization domain or when a dimerization domain was present. The presence of a tetramerization domain induced formation of tetramers only, and resulted in the highest specific activity. While it will be of interest to study the oligomeric state of the HN proteins of hPIV1, SeV, hPIV3 with and without oligomerization domains, these studies were not performed due to low expression levels for most of these proteins. Paramyxovirus virions and HN-NPs exclusively bound to α2-3-linked Sia receptors ([60] and this study). In addition, NDV HN-NPs were motile on a receptor-coated surface in a sialidase-dependent manner just like virions [60], thereby cleaving sialoglycans until a certain desialylation threshold is reached and particles are released. This desialylation threshold was independent of particle concentration, but logically reached faster (i.e., self-elution was faster) with higher HN-NP concentrations. Thus, just as virions, the motility of HN-NPs is reminiscent of a burnt-bridge ratchet or lawnmower molecular motor model [21,22]. While the HN-NP system showed nanoparticle motility to be independent of other viral proteins, it will be of interest to analyze whether presence of the F protein may affect the receptor interaction of the HN-NPs, in view of the balance that exists between the different functions of HN, which include F stabilization, receptor binding, F activation and receptor-cleavage (reviewed in [91]).

Some, but not all, sialoglycan-binding paramyxoviruses appear to contain a 2SBS. The presence of a 2SBS in NDV is well accepted, based on structural data [25,34] and the ability of NDV to interact with receptors in the presence of site I inhibitors (zanamivir or BCX2798) [33,92]. In agreement with these results, we observed efficient binding of NDV virions and HN-NPs in the presence of BCX2798 ([60] and this study), while this BCX2798-independent binding was severely inhibited by several substitutions in the 2SBS (this study). For human PIVs, the presence of a functional 2SBS is less well-established. Under selective pressure from a NA inhibitor, a hPIV3 variant with a H552Q substitution emerged, displaying increased receptor-binding and partial resistance to zanamivir in a red blood cell elution assay, while its sialidase activity was not changed [37,38], leading to a conclusion of a functional 2SBS. However, in a later study it was reported that, rather than H552Q, substitution N523D in HN of hPIV3, which removed a glycosylation site from the dimer interface, restored binding via a 2SBS [36]. Also for hPIV1 HN, removal of a glycan appeared to uncover a 2SBS [35]. In these latter two studies, removal of the N-glycan resulted in HN-receptor binding that was insensitive for site I inhibitor BCX2798 and the related compound BCX2855. For SeV, binding and cleavage were concluded to occur via independent sites based on a temperature-sensitive and antigenic variants [39,40]. Our results do not indicate the existence of a functional 2SBS in the hPIV1, SeV and hPIV3 viruses studied here, as BCX2798 completely inhibited binding of nanoparticles carrying their HN proteins. We cannot exclude, however, that BCX2798 binds to a putative 2SBS in these HN proteins, although that seems unlikely based on previous studies using this or a related compound with hPIV1 and hPIV3 [35,36].

The HN-NP system provides an alternative approach for investigating receptor interactions of viruses which are difficult to culture, including clinal isolates without adaptive mutations or mutant viruses with a lethal phenotype. As an example, we compared the receptor-interaction of an hPIV3 clinical isolate (CI) versus a laboratory-adapted strain (LS). hPIV3 adapts to cell culture via increased receptor binding and reduced receptor-cleavage of HN, along with increased fusion activity, thereby highlighting the different requirements for optimal replication and spread *in vitro* versus *in vivo* [46,48,49]. Our nanoparticle studies recapitulated the different receptor binding, with hPIV3 CI showing a lower initial binding rate in BLI and a higher sialidase activity (specific activity, K_cat_ and K_cat_/K_m_) than hPIV3 LS, indicating hPIV3 CI is favoring short-lived receptor engagement (Fig 8). The disparate preferences in receptor-binding and -cleavage functionality between hPIV3 CI and LS were shown to be largely modulated by the key residue 556 in agreement with previous studies [14,49] (S10 Fig), as evidenced by the CI-like behavior exhibited by the LS-D556N mutant HN-NPs (Fig 8).

To exemplify the use of the nanoparticle platform to study potential lethal mutations, we analyzed a set of substitutions in the catalytic site of NDV HN. The importance of conserved residues in site I for receptor binding and cleavage has been studied previously (summarized in S2 Table) [79–82,84,86], including several substitutions that were studied here. In agreement with our results, substitution of R498 was shown to affect receptor cleavage much more than receptor binding [81,82], while E401 [81] and D198 [79,80,82] were important for both activities. In the background of hPIV3 HN, however, substitution of the residue corresponding to D198 (D216) was shown to negatively affect sialidase activity and triggering of F, but not receptor binding *per se* [44]. Varying results in different studies were obtained after substitution of residues at position 174, 416 and 526, which may be explained (in part) by different HN proteins and assays (hemadsorption assays vs HN-NPs) used in the different studies ([79–82] and this study). While several substitutions resulted in complete loss of receptor-cleavage activity (Fig 5C, E401A, R416A, R498A and Y526A), some of the mutant proteins retained the capacity to bind to receptors, which was particularly observed for substitutions R416A and R498A (Fig 5E). The significantly reduced receptor binding of HN R498A in the presence of BCX2798 indicates that this protein binds via the catalytically-dead site I. In contrast, BCX2798 did not significantly affect binding of HN R416A, which may be explained by the inhibitor not being able to bind the catalytic site of this protein, or that this HN binds mainly via the 2SBS. The results furthermore indicate that absence of cleavage may be caused by reduced substrate binding and thus cleavage (E401A) as well as by absence of cleavage after binding (R416A and R498A). The total lack of sialidase activity and binding for E401A HN may be attributed to a crucial role of this residue in receptor binding and thus cleavage, or alternatively indicate a general folding defect. It will be of interest to analyze to what extent the substitutions studied here may affect the secondary structure of HN.

Substitutions in the site I may not only affect receptor cleavage and binding activity via this site, but also activity of the 2SBS. For example, substitutions R498A and Y526A not only negatively affected receptor cleavage, but while HN-NPs were still able to bind in the absence of BCX2798, binding was severely affected via the 2SBS (in the presence of BCX2798) compared to WT (Fig 5). Vice versa, several substitutions in the 2SBS also affected cleavage kinetics of 4-MUNANA and binding in the absence of BCX2798 (e.g., F156A, V157A and F553A). Similar long-range effects between site I and the 2SBS of NDV HN have also been noted in previous studies [81,82]. Of note, residue Y526 located in the catalytic site is connected via a loop to residues F553 and L552 in the 2SBS (S11 Fig). Therefore, alterations in either site could affect the other site, e.g., by shifting the loop. Others suggested that receptor binding via site I induces a conformational change in the HN dimer interface, thereby impacting the binding activity of the 2SBS [86] and affecting fusion activation [34]. Intramolecular long-range effects have previously also been described to play an important role in IAV neuraminidase as well as other viral attachment proteins [93–95]. The 2SBS in NDV HN may contribute to cleavage of multivalent substrates similarly as IAV NA [96]. However, the long-range effects between the site I and 2SBS in NDV HN currently do not allow us to prove this experimentally.

HN exhibited greater enzymatic activity towards a receptor-coated surface when present on nanoparticles compared to an equivalent amount of soluble HN (Fig 4B), which can most likely be attributed to local concentration effects. Likewise, neuraminidase activity of influenza A virus particles is influenced by HA-receptor binding [20,97]. Cooperativity has also been described for other multivalent enzymes [98]. Multivalent dynamic receptor interactions were difficult to predict from Michaelis-Menten kinetics using soluble enzymes. This phenomenon can be attributed to the multivalent presentation of receptors in conjunction with multivalent particles. For instance, substitution S222A in NDV HN, which had an approximately two-fold negative effect on binding at the 2SBS (in presence of BCX2798), resulted in a higher K_cat_ and K_m_ value and a two-fold lower K_cat_/K_m_. How this relates to the approximately similar initial binding rate in the absence of BCX2798 and an overall lower particle-surface interaction (as evidenced by an approximately 3-fold lower area under the curve) is currently not clear. Likewise, it is not immediately clear why hPIV1 and SeV HN proteins that differ in their K_cat_ and K_m_ (both being 3-fold higher for SeV) but have a similar K_cat_/K_m_ and specific activity, differ in their interaction with a receptor-coated surface when coupled to nanoparticles. To what extent these differences in HN-receptor interactions between SeV and hPIV3 relate to adaptation to human- and mouse-specific sialoglycan profiles remains to be determined.

In conclusion, our study introduces a novel platform for investigating multivalent virus-receptor dynamics of paramyxoviruses, which probably is also applicable to other sialoglycan-binding viruses including IAV and coronaviruses. This platform will facilitate analysis of these interactions particularly for viruses that are difficult propagate and for mutant attachment/receptor-destroying glycoproteins without the risk of performing gain-of-function research. Application of this platform allowed us to elucidate differences in the dynamic interactions of human and animal paramyxoviruses, and to study in more detail long-range interactions between the catalytic site and the 2SBS of NDV HN. Analysis of these multivalent interactions will be important to elucidate characteristics of animal versus human viruses and determinants of host tropism. In addition, nanoparticles of different sizes and shapes could be decorated with viral glycoproteins to analyze the effect thereof on receptor-interactions and mobility by BLI or live microscopy. On the other hand, such nanoparticles might also be used for the delivery of drugs over mucosal surfaces, similarly as was described previously [99].

## Material and methods

### Cells and viruses

Lewis lung carcinoma-monkey kidney (LLC-MK2) cells were cultured in Dulbecco’s modified Eagle’ s medium (DMEM) (Thermo Fisher Scientific), supplemented with 10% fetal bovine serum (Biowest), 1 mM sodium pyruvate (Gibco), 100 IU/ml penicillin, and 100 IU/ml streptomycin (Lonza), at 37°C in a humidified CO_2_ incubator. The cell lines tested negative for mycoplasma. NDV (Nobilis ND Clone-30) was purchased from MSD Animal Health and used directly in the experiments. The following reagent was obtained through BEI Resources, NIAID, NIH: Sendai Virus (formerly Parainfluenza Virus 1, Sendai), NR-3227; Human Parainfluenza Virus 3, NIH 47885, NR-3233. Human parainfluenza virus type 1 (strain Washington/20993/1964, GenBank accession no. AF016280) was purchased from ViraTree. hPIV1, hPIV3 and SeV were propagated in LLC-MK2 cells using Opti-MEM (Thermo Fisher Scientific). 1 μg/ml TPCK trypsin (Sigma-Aldrich) was additionally added for hPIV1 and SeV. The viruses were aliquoted and stored at -80°C until use.

### Expression and purification of recombinant protein

Proteins were expressed in Freestyle 293-F cells (Gibco) that were maintained in Freestyle 293 expression medium (37°C). Human codon-optimized ectodomain encoding cDNAs (GenScript) HN of NDV (GenBank accession no. CAB51326.1), SeV (GenBank accession no. QED12413.1), hPIV1 (GenBank accession no.AAC23946.1), hPIV3 LS (GenBank accession no. AET35008.1) and hPIV3 CI GenBank accession no. AOO33557.1) were obtained from GenScript. The HN cDNAs encode identical ectodomains as the viruses used. These cDNAs were cloned into a pFRT expression plasmid (Thermo Fisher Scientific) fused at their 5’ends to sequences encoding a GL signal peptide, a 6xHistidine tag (6His) and a Twin-Strep tag. The dimer and tetramer constructs of HNs were further modified by incorporating GCN4IL dimerization domain [71] and tetrabrachion tetramerization domain-encoding sequences after the Strep tag encoding sequence, respectively. All proteins were expressed transiently in HEK-293F (CVCL-6642), and secreted proteins were purified from supernatants using strep-tactin beads (IBA) following the manufacturer’s protocol. The purified proteins were quantified using quantitative densitometry of GelCode Blue (Thermo Fisher Scientific)-stained protein gels which also contained bovine serum albumin (BSA) standards, and the generated images were analyzed with an Odyssey imaging system (LI-COR). The conformation of NDV monomer, dimer and tetramer constructs was analyzed by size exclusion chromatography (ӒKTA pure chromatography system) Superdex 200 Increase 10/300 GL column. Different HN mutants with single-site residue substitutions were generated using Q5 High-fidelity DNA polymerase (NEB) by site-directed mutagenesis and confirmed by sequencing.

### Coupling of glycoproteins to Ni-NTA nanoparticles

The gold Ni-NTA nanoparticles (30–100 nm) were purchased from Cytodiagnostics, while dextran iron oxide composite Ni-NTA nanoparticles (130 nm, 250 nm) were purchased from Micromod. A standard coupling ratio of HN: 130 nm nanoparticles of 0.45 μg: 2.63 x 10^10^ particles (manufacturer’s specifications, corresponding to 7.43 x 10^8^ particles as determined by NTA, S1 Table) in 100 μl PBS^+/+^ was used and defined as standard coupling amount. Of note, the concentration of nanoparticles as provided by the manufacturers deviated from the values as determined by NTA (S1 Table). Coupling was performed at 4°C overnight. Subsequently, the HN-nanoparticle preparation was centrifuged at 2,000 rpm for 10 minutes, and the supernatant was carefully aspirated to remove any free proteins, followed by filling with the same volume (100 μl) of PBS^+/+^. 15 μl of the resulting coupled HN-nanoparticles preparation was used in the subsequent BLI experiments. Remaining HN-NPs were collected by centrifugation, taken up in 4x Laemmli sample buffer (BioRad), heated to 95°C for 10 min, and electrophoresed in 6–12% continuous SDS–PAGE gel (Bio-Rad) followed by transfer onto a PVDF membrane (Bio-Rad). Presence of HN proteins were probed using a StrepMAB-Classic conjugated to HRP (IBA) targeting the Strep-tag. The western blot images were captured and quantified using the Odyssey imaging system (LI-COR).

### Negative staining of the HN-Ni-NTA nanoparticles

Gold 100 nm Nanoparticles were diluted to 3.91 x 10^10^ particles/mL (manufacturer’s specifications) immediately prior to adsorption to glow-discharged carbon-coated copper grids. The sample was allowed to absorb for 30 seconds prior to two times wash with sterile water, followed by staining with 2% uranyl acetate solution. Representative micrographs were recorded on a 120 kV FEI Talos L120C with a 4k×4k Ceta 16M CCD camera at 22,000 or 92,000 nominal magnification.

### Sialidase assays

The specific sialidase activity was assessed using 2’-(4-Methylumbelliferyl)-α-d-N-acetylneuraminic acid sodium salt (4-MUNANA, Sigma-Aldrich) as described previously [60]. The 4-MUNANA substrate generates a fluorescent product, 4-methylumbelliferone (4-MU), upon cleavage by HN enzymes, allowing for quantitative measurement of fluorescence intensity. To determine the specific activity, 1 μg of proteins was subjected to 2-fold serial dilutions in PBS^+/+^ buffer in a flat-bottom 96-well black plate (Greiner Bio-One). Subsequently, 50 μl of 4-MUNANA substrate (200 μM) was added to each well. Following incubation at 37°C for 60 minutes in the 96-well plate, the reaction was terminated by adding 190 μl of stop solution (0.1 M glycine, 25% ethanol, pH 10.7). The fluorescence intensity was measured using an excitation wavelength of 360 nm and an emission wavelength of 450 nm. To determine the initial rates (V) of HNs, equal amounts of proteins were incubated with increasing concentrations of the 4-MUNANA substrate (0–5 mM) in PBS^+/+^ buffer. Fluorescence was monitored at 60-second intervals for 60 min at 37°C. A standard curve was generated for each experiment using 4-MU diluted in PBS^+/+^ buffer, ranging from 0.1 μM to 625 μM. The recorded fluorescence was corrected for the background and converted to 4-MU concentration (μM) for further analysis. Time course data for each concentration of the 4-MUNANA substrate were examined for linearity by linear regression analysis. Only data with R^2^>0.98 were included in subsequent analysis. The Michaelis constant K_m_ and maximal reaction velocity V_max_ of the HNs were determined by fitting the data to the Michaelis–Menten model using GraphPad Prism 9.3.2. Furthermore, the K_cat_ values were calculate based on the enzyme concentration ([E]) and V_max_ (V_max_ = K_cat_ [E]).

### Hemagglutination assay

Human red blood cells (hRBCs; provided by Sanquin) were washed with cold PBS with Ca^2+^ and Mg^2+^ (PBS^+/+^) until the supernatant became clear and then suspended to 50% in PBS^+/+^. The HN-conjugated 130 nm Ni-NTAs nanoparticles (HN-NPs) were then 3-fold serially diluted and mixed with 1:1 with hRBC (0.5% in PBS^+/+^). Hemagglutination was assessed after 3 h incubation at 4°C, and hemagglutinating units (HAU) were calculated for each HN-NP.

### BLI assay

Phosphate-buffered saline with Ca^2+^ and Mg^2+^ (PBS^+/+^, Lonza) served as the buffer for all Bio-Layer Interferometry (BLI) experiment. Biotinylated synthetic glycans were generously provided by Geert-Jan Boons (Department Chemical Biology and Drug Discovery, Utrecht University) (3’S(LN)_3_ and 6’S(LN)_3_)) [100]. Standard streptavidin sensors (SA, Pall-ForteBio) were employed for BLI “dip and read” analyses, following a similarly procedure as described previously [74]. Briefly, the streptavidin (SA) sensors were loaded with specific synthetic glycans and then incubated in PBS^+/+^ until a stable baseline was achieved. Next, receptor-loaded sensors were incubated in PBS^+/+^ containing proteins or protein-Ni-NTA nanoparticles for the designated time to generate a binding curve. When indicated, 0.5 mM BCX2798(4-azido-5-isobutyrylamino-2,3-didehydro-2,3,4,5-tetradeoxy-d-glycero-d-galacto-2-nonulopy-ranosic acid, synthesized in house [60]) was added. To remove bound HN-NPs or lectins, the sensors were regenerated by three 5 s washes in 10 mM Tris/Glycine buffer (pH 2.0), which preserves the binding of biotinylated receptors [20]. The regeneration step was specifically performed for the analysis of MAL I and ECA binding. All experiments were performed 3 to 5 times at 30°C, utilizing at least two independently generated protein stocks. The negative binding curve observed in BLI results from the large size of the nanoparticles in comparison to soluble proteins and is also observed for enveloped virions and other vesicles [60,74,77]. Representative experiments are depicted and all correlation analyses were carried out using GraphPad Prism 9.3.2.

### Nanoparticle tracking analysis (NTA)

The NanoSight NS300 instrument (Malvern) was used to quantify virus particle/nanoparticle numbers as described previously [60,101]. In brief, the virus solution was appropriately diluted in PBS^+/+^ for Nanoparticle Tracking Analysis (NTA) analysis. The NanoSight NS300 instrument captured five 60-second sample video per analysis. These videos were subsequently analyzed in the Nanoparticle Tracking analysis 3.0 software, which provide a quantitative information on nanoparticle numbers. All measurements were performed at 19°C. Each sample was analyzed twice, and mean values were utilized. It should be noted that 1.0 x 10^10^ virions in the virus preparations, as determined by NTA, typically corresponded to approximately 1.0 x 10^8^ TCID_50_ units of hPIV1, hPIV3, SeV and NDV.

### Statistical analysis

A P value equal or less than 0.05 was considered significant, * P≤0.05, ** P≤0.01, *** P≤0.001 and **** P < 0.0001. ns (not significant), P > 0.05. GraphPad Prism version 9.3.2 (GraphPad Software) was used for data analysis and statistics.

## Supporting information

S1 Fig Analysis of HN-NPs of different sizes in BLI. **(A)** Schematic representation of the production and analysis of HN-NPs. **(B)** Based on the manufacture’s specification, 0.45μg HN coupled to 1.44 x 10^9^ gold nanoparticles were used in the BLI assay using 3’S(LN)_3_ receptor in the presence or absence of BCX2798. **(C)** At the same time, 9.60 x 10^9^ gold HN-NPs were collected by centrifugation and analyzed by Western-blot analysis. Lane indicated with HN contains the input amount of HN protein. **(D)** Similarly, 0.45μg HNs coupled to 2.63 x 10^10^ dextran iron oxide composite particles (manufacture’s specification) were used in the BLI assay using 3’S(LN)_3_ receptor in the presence or absence of BCX2798. **(E)** At the same time, 1.75 x 10^11^ HN-NPs were collected by centrifugation and analyzed by Western-blot analysis. Lane indicated with HN contains the input amount of HN protein. Based on the specifications of the manufacturers, approximately 10-fold higher particle numbers were used for the dextran iron oxide composite particles than for the gold particles. However, based on nanoparticle tracking analysis (NTA) the particle numbers used were quite similar, particularly for the 100 and 130 nm particles (S1 Table) and in the same range as the virion number. S1A Fig created with Biorender.com.
(DOCX)

S2 Fig Comparison of catalytic site inhibitors BCX2798 and Zanamivir inhibition in the BLI assay. Streptavidin sensors were loaded to saturation with 3’S(LN)_3_. Subsequently, the sensor was incubated with (A) 1.0 x 109 NDV virions, (B) 0.45 μg HN coupled to 4.41 x 10^8^ gold nanoparticles in the absence or presence of catalytic site inhibitor BCX2798 (0.5 mM) or Zanamivir (0.5 mM or 10mM). Particle numbers indicated are according to NTA analysis (also see S1 Table).
(DOCX)

S3 Fig Optimization of HN-NP coupling ratio for BLI analysis. (A) Binding of HN-NPs to sensors coated with 3’S(LN)_3_ was performed similarly as described in the Fig 4 legend. Different amounts of HN (μg; indicated in the figure) were coupled to 4.95 x 10^9^ Ni-NTA nanoparticles (130 nm, nanoparticle number according to NTA analysis), followed by standard wash step as described in Methods, then 7.43 x 10^8^ HN-NPs were used to associate in the BLI analysis. Coupling of 3 ug HN corresponds with the standard coupling condition. (B) Different amounts of HN (μg) were coupled to 4.95 x 10^9^ Ni-NTA nanoparticles, then samples were directly centrifuge in 2,000 rpm for 10 min, supernatant (SUP) and HN-NPs (NPs) were collected separately and subject to Western blot analysis. Same amounts of HNs as used for coupling were used as control (bottom lane). Nanoparticle numbers indicated here are according to NTA analysis, see also S1 Table.
(DOCX)

S4 Fig Nanoparticle tracking analysis (NTA) of 130nm nanoparticles with or without HN present. An example of a NTA experiment using the NanoSight NS300 instrument is shown. The black line corresponds empty 130nm Ni-NTA nanoparticles, while the red curve represents the same particles coupled with HN (HN-NPs). The NTA shows that the size of the major peak of the empty particles corresponds to 114 nm, while it is bigger for the HN-NPs (134 nM) in agreement with them being coated with HN. Only some minor larger peaks are observed indicating only minor aggregation of these particles in solution. Particle concentration (in 10^10^ particles/ml) and diameter (in nm) are graphed on the Y- and X-axis, respectively. The concentrations shown are the mean of 5 measurements ± SD.
(DOCX)

S5 Fig HN-NPs bind α2-3Sialoglycan-, but not α2-6Sialoglycan-coated sensors. (A) Schematic representation of the biotinylated sialoglycans used in this study. 3’S(LN)_3_: Neu5Acα2-3Galβ1-4GlcNAcβ1-3Galβ1-4GlcNAcβ1-3Galβ1-4GlcNAc, 6’S(LN)_3_: NeuAcα2-6Galβ1-4GlcNAcβ1-3Galβ1-4GlcNAcβ1-3Galβ1-4GlcNAc. HN-NP binding curves were generated similarly as described in the Fig 5 legend using 3’S(LN)_3_ or 6’S(LN)_3_ for (B) NDV HN-NPs (with or without BCX2798), (C) hPIV1 and SeV HNs-NPs, and (D) hPIV3 CI, LS and LS-D556N HNs-NPs. S5A Fig created with Biorender.com.
(DOCX)

S6 Fig No binding of empty nanoparticles to the sensors. 7.43 x 10^8^ empty NPs (130 nm) were allowed to interact with 3’S(LN)_3_-, 6’S(LN)_3_-, or biotin-coated sensors. As a negative control, PBS was taken along. Nanoparticle numbers indicated here are according to NTA analysis, see also S1 Table.
(DOCX)

S7 Fig Sialidase activity of coupled and unconjugated HN. The sialidase activity of NDV HN-NPs (130nm, 7.43 x 10^8^ HN-NPs) or corresponding amount of soluble HNs (assuming 100% coupling efficiency) were determined by applying the 4-MUNANA fluorometric assay under standard conditions. Nanoparticle numbers indicated here are according to NTA analysis, see also S1 Table.
(DOCX)

S8 Fig Hemagglutination analysis of the soluble HNs and HN-NPs. Hemagglutination was performed starting with 7.43 x 10^8^ HN-NPs (130 nm, standard coupling) in 1st well or with the corresponding amount of soluble HNs (assuming a 100% coupling efficiency) using human erythrocytes. Nanoparticle numbers indicated here are according to NTA analysis, see also S1 Table.
(DOCX)

S9 Fig Correlation analysis of NDV HN WT and mutant proteins. Data were normalized to the max value of each row. The heat map was drawn using ChiPlot (https://www.chiplot.online/) (accessed on 09 January 2024).
(DOCX)

S10 Fig Sequence alignment of the HN proteins of the hPIV3 lab strain (LS) and clinical isolate (CI) used in this study. Differences between LS and CI sequences are highlighted in yellow, while the residues of the primary receptor binding site (site I) are highlighted in magenta. Residue 556 substituted in this study is indicated. hPIV3 LS (GenBank accession no. AET35008.1), hPIV3 CI GenBank accession no. AOO33557.1).
(DOCX)

S11 Fig Cartoon representation of the NDV HN Sia binding pockets. Residues located in site I are colored in deepblue, and residues in site II are colored cyan. Residues linked with the same loop between site I and site II are shown in hotpink (PDB ID: 1USR). The thiosialoside is shown with yellow bonds.
(DOCX)

S1 Table Nanoparticle concentration and HN tetramers density on single particles. (DOCX)

S2 Table Summary of mutagenesis studies performed for site I and site II/interface of NDV HN. (DOCX)

S3 Table Statistical analysis of initial binding rates of NDV HN-NPs in the absence or presence of BCX2798 (based on results shown in Figs 5 and 6). (DOCX)

S1 Raw data Raw data Wu *et al*. (XLSX)
