# Supplementary material for: Unraveling dynamics of paramyxovirus-receptor interactions using nanoparticles displaying hemagglutinin-neuraminidase
Source: PLoS Pathog. 2024 Jul 25;20(7):e1012371. doi: 10.1371/journal.ppat.1012371 (PMC11302929; doi:10.1371/journal.ppat.1012371)
Supplement: S1 Fig — (A) Schematic representation of the production and analysis of HN-NPs. (B) Based on the manufacture’s specification, 0.45μg HN coupled to 1.44 x 10^9^ gold nanoparticles were used in the BLI assay using 3’S(LN)3 receptor in the presence or absence of BCX2798. (C) At the same time, 9.60 x 10^9^ gold HN-NPs were collected by centrifugation and analyzed by Western-blot analysis. Lane indicated with HN contains the input amount of HN protein. (D) Similarly, 0.45μg HNs coupled to 2.63 x 10^10^ dextran iron oxide composite particles (manufacture’s specification) were used in the BLI assay using 3’S(LN)3 receptor in the presence or absence of BCX2798. (E) At the same time, 1.75 x 10^11^ HN-NPs were collected by centrifugation and analyzed by Western-blot analysis. Lane indicated with HN contains the input amount of HN protein. Based on the specifications of the manufacturers, approximately 10-fold higher particle numbers were used for the dextran iron oxide composite particles than for the gold particles. However, based on nanoparticle tracking analysis (NTA) the particle numbers used were quite similar, particularly for the 100 and 130 nm particles (S1 Table) and in the same range as the virion number. S1A Fig created with Biorender.com. (DOCX) [file ppat.1012371.s001.docx]

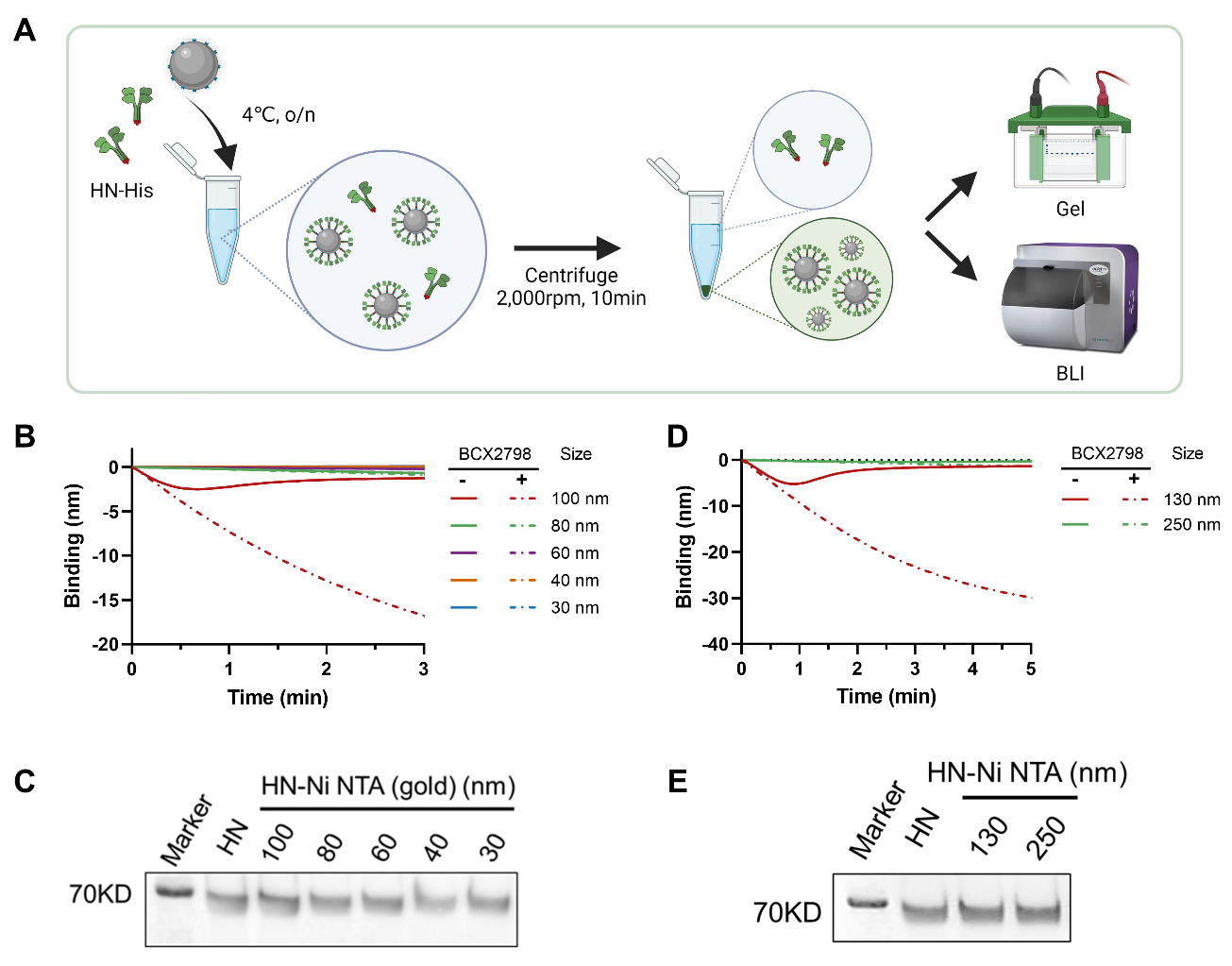


**S1 Fig. Analysis of HN-NPs of different sizes in BLI.** **(A)** Schematic representation of the production and analysis of HN-NPs. **(B)** Based on the manufacture’s specification, 0.45μg HN coupled to 1.44 x 10^9^ gold nanoparticles were used in the BLI assay using 3’S(LN)_3_ receptor in the presence or absence of BCX2798. **(C)** At the same time, 9.60 x 10^9^ gold HN-NPs were collected by centrifugation and analyzed by Western-blot analysis. Lane indicated with HN contains the input amount of HN protein. **(D)** Similarly, 0.45μg HNs coupled to 2.63 x 10^10^ dextran iron oxide composite particles (manufacture’s specification) were used in the BLI assay using 3’S(LN)_3_ receptor in the presence or absence of BCX2798. **(E)** At the same time, 1.75 x 10^11^ HN-NPs were collected by centrifugation and analyzed by Western-blot analysis. Lane indicated with HN contains the input amount of HN protein. Based on the specifications of the manufacturers, approximately 10-fold higher particle numbers were used for the dextran iron oxide composite particles than for the gold particles. However, based on nanoparticle tracking analysis (NTA) the particle numbers used were quite similar, particularly for the 100 and 130 nm particles (S1 Table) and in the same range as the virion number.
