# Supplementary material for: Unraveling dynamics of paramyxovirus-receptor interactions using nanoparticles displaying hemagglutinin-neuraminidase
Source: PLoS Pathog. 2024 Jul 25;20(7):e1012371. doi: 10.1371/journal.ppat.1012371 (PMC11302929; doi:10.1371/journal.ppat.1012371)
Supplement: S2 Fig — Streptavidin sensors were loaded to saturation with 3’S(LN)3. Subsequently, the sensor was incubated with (A) 1.0 x 10^9^ NDV virions, (B) 0.45 μg HN coupled to 4.41 x 10^8^ gold nanoparticles in the absence or presence of catalytic site inhibitor BCX2798 (0.5 mM) or Zanamivir (0.5 mM or 10mM). Particle numbers indicated are according to NTA analysis (also see S1 Table). (DOCX) [file ppat.1012371.s002.docx]

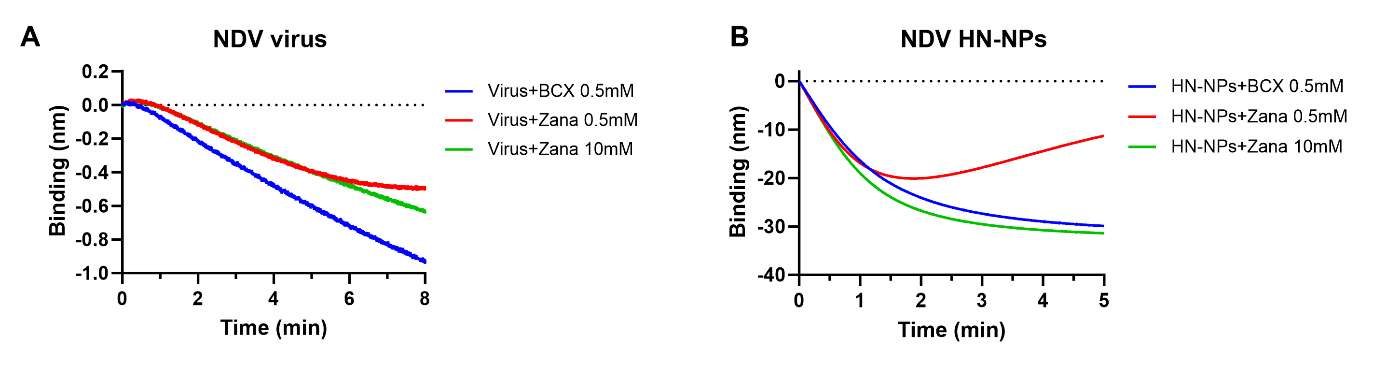


**S2 Fig. Comparison of catalytic site inhibitors BCX2798 and Zanamivir inhibition in the BLI assay.** Streptavidin sensors were loaded to saturation with 3’S(LN)_3_. Subsequently, the sensor was incubated with (A) 1.0 x 109 NDV virions, (B) 0.45 μg HN coupled to 4.41 x 10^8^ gold nanoparticles in the absence or presence of catalytic site inhibitor BCX2798 (0.5 mM) or Zanamivir (0.5 mM or 10mM). Particle numbers indicated are according to NTA analysis (also see S1 Table).
