# Supplementary material for: Unraveling dynamics of paramyxovirus-receptor interactions using nanoparticles displaying hemagglutinin-neuraminidase
Source: PLoS Pathog. 2024 Jul 25;20(7):e1012371. doi: 10.1371/journal.ppat.1012371 (PMC11302929; doi:10.1371/journal.ppat.1012371)
Supplement: S3 Fig — (A) Binding of HN-NPs to sensors coated with 3’S(LN)3 was performed similarly as described in the Fig 4 legend. Different amounts of HN (μg; indicated in the figure) were coupled to 4.95 x 10^9^ Ni-NTA nanoparticles (130 nm, nanoparticle number according to NTA analysis), followed by standard wash step as described in Methods, then 7.43 x 10^8^ HN-NPs were used to associate in the BLI analysis. Coupling of 3 ug HN corresponds with the standard coupling condition. (B) Different amounts of HN (μg) were coupled to 4.95 x 10^9^ Ni-NTA nanoparticles, then samples were directly centrifuge in 2,000 rpm for 10 min, supernatant (SUP) and HN-NPs (NPs) were collected separately and subject to Western blot analysis. Same amounts of HNs as used for coupling were used as control (bottom lane). Nanoparticle numbers indicated here are according to NTA analysis, see also S1 Table. (DOCX) [file ppat.1012371.s003.docx]

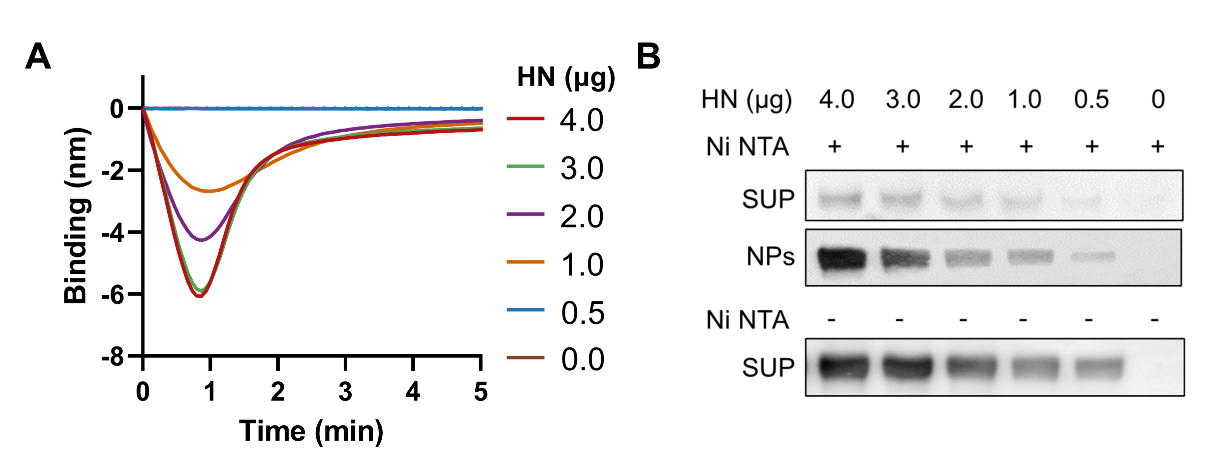


**S3 Fig. Optimization of HN-NP coupling ratio for BLI analysis.** (A) Binding of HN-NPs to sensors coated with 3’S(LN)_3_ was performed similarly as described in the Fig 4 legend. Different amounts of HN (μg; indicated in the figure) were coupled to 4.95 x 10^9^ Ni-NTA nanoparticles (130 nm, nanoparticle number according to NTA analysis), followed by standard wash step as described in Methods, then 7.43 x 10^8^ HN-NPs were used to associate in the BLI analysis. Coupling of 3 ug HN corresponds with the standard coupling condition. (B) Different amounts of HN (μg) were coupled to 4.95 x 10^9^ Ni-NTA nanoparticles, then samples were directly centrifuge in 2,000 rpm for 10 min, supernatant (SUP) and HN-NPs (NPs) were collected separately and subject to Western blot analysis. Same amounts of HNs as used for coupling were used as control (bottom lane). Nanoparticle numbers indicated here are according to NTA analysis, see also S1 Table.
