# Supplementary material for: Unraveling dynamics of paramyxovirus-receptor interactions using nanoparticles displaying hemagglutinin-neuraminidase
Source: PLoS Pathog. 2024 Jul 25;20(7):e1012371. doi: 10.1371/journal.ppat.1012371 (PMC11302929; doi:10.1371/journal.ppat.1012371)
Supplement: S4 Fig — An example of a NTA experiment using the NanoSight NS300 instrument is shown. The black line corresponds empty 130nm Ni-NTA nanoparticles, while the red curve represents the same particles coupled with HN (HN-NPs). The NTA shows that the size of the major peak of the empty particles corresponds to 114 nm, while it is bigger for the HN-NPs (134 nM) in agreement with them being coated with HN. Only some minor larger peaks are observed indicating only minor aggregation of these particles in solution. Particle concentration (in 1010 particles/ml) and diameter (in nm) are graphed on the Y- and X-axis, respectively. The concentrations shown are the mean of 5 measurements ± SD. (DOCX) [file ppat.1012371.s004.docx]

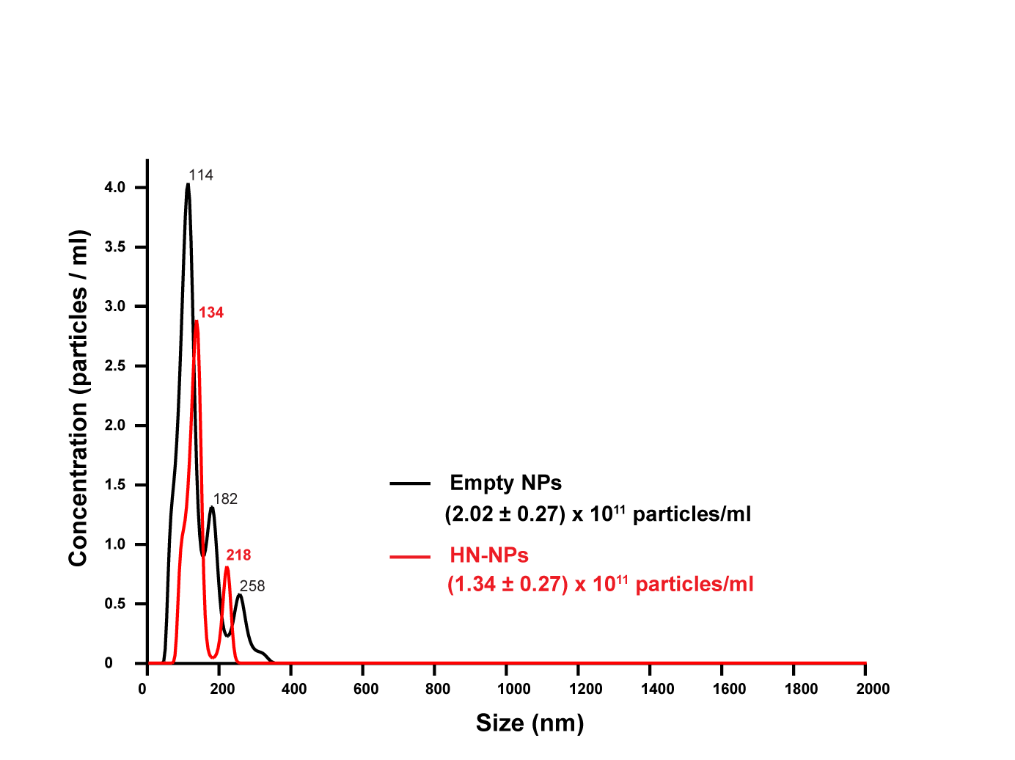


**S4 Fig. Nanoparticle tracking analysis (NTA) of 130nm nanoparticles with or without HN present.** An example of a NTA experiment using the NanoSight NS300 instrument is shown. The black line corresponds empty 130nm Ni-NTA nanoparticles, while the red curve represents the same particles coupled with HN (HN-NPs). The NTA shows that the size of the major peak of the empty particles corresponds to 114 nm, while it is bigger for the HN-NPs (134 nM) in agreement with them being coated with HN. Only some minor larger peaks are observed indicating only minor aggregation of these particles in solution. Particle concentration (in 10^10^ particles/ml) and diameter (in nm) are graphed on the Y- and X-axis, respectively. The concentrations shown are the mean of 5 measurements ± SD.
