# Supplementary material for: Unraveling dynamics of paramyxovirus-receptor interactions using nanoparticles displaying hemagglutinin-neuraminidase
Source: PLoS Pathog. 2024 Jul 25;20(7):e1012371. doi: 10.1371/journal.ppat.1012371 (PMC11302929; doi:10.1371/journal.ppat.1012371)
Supplement: S5 Fig — (A) Schematic representation of the biotinylated sialoglycans used in this study. 3’S(LN)3: Neu5Acα2-3Galβ1-4GlcNAcβ1-3Galβ1-4GlcNAcβ1-3Galβ1-4GlcNAc, 6’S(LN)3: NeuAcα2-6Galβ1-4GlcNAcβ1-3Galβ1-4GlcNAcβ1-3Galβ1-4GlcNAc. HN-NP binding curves were generated similarly as described in the Fig 5 legend using 3’S(LN)3 or 6’S(LN)3 for (B) NDV HN-NPs (with or without BCX2798), (C) hPIV1 and SeV HNs-NPs, and (D) hPIV3 CI, LS and LS-D556N HNs-NPs. S5A Fig created with Biorender.com. (DOCX) [file ppat.1012371.s005.docx]

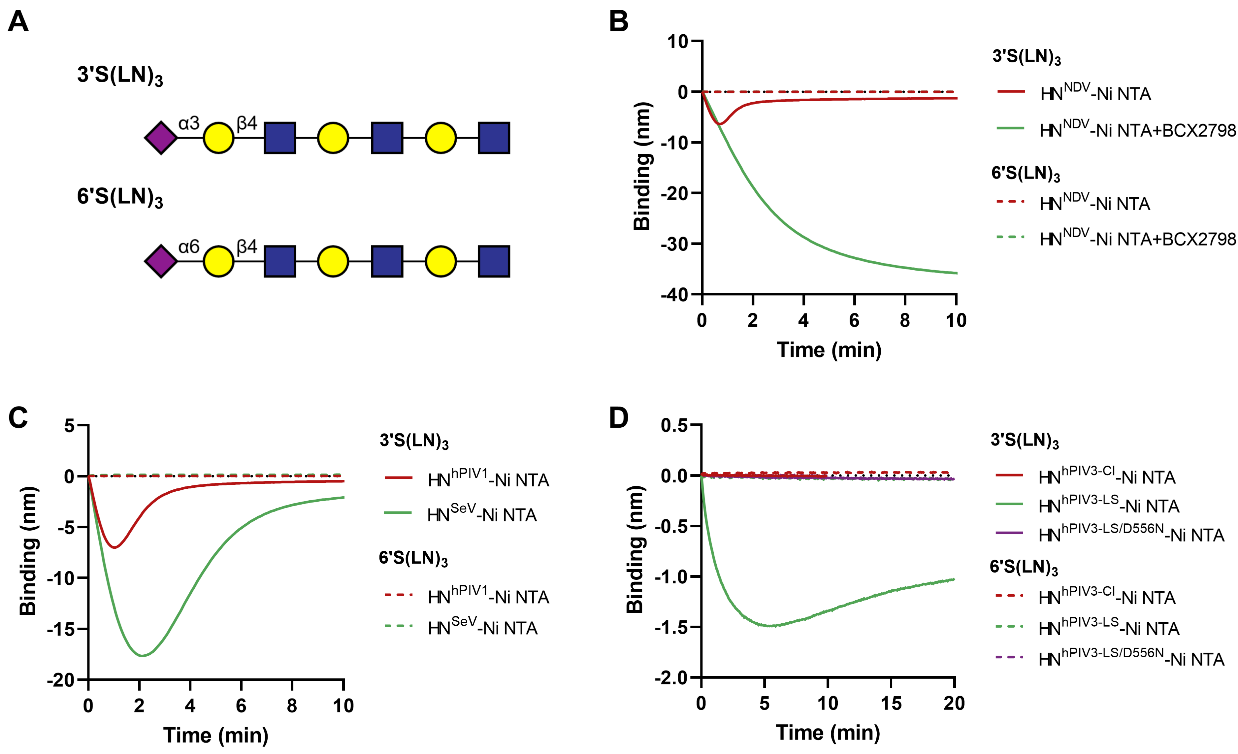


**S5 Fig. HN-NPs bind α2-3Sialoglycan-, but not α2-6Sialoglycan-coated sensors.**  (A) Schematic representation of the biotinylated sialoglycans used in this study. 3’S(LN)_3_: Neu5Acα2-3Galβ1-4GlcNAcβ1-3Galβ1-4GlcNAcβ1-3Galβ1-4GlcNAc, 6’S(LN)_3_: NeuAcα2-6Galβ1-4GlcNAcβ1-3Galβ1-4GlcNAcβ1-3Galβ1-4GlcNAc. HN-NP binding curves were generated similarly as described in the Fig 5 legend using 3’S(LN)_3_ or 6’S(LN)_3_ for (B) NDV HN-NPs (with or without BCX2798), (C) hPIV1 and SeV HNs-NPs, and (D) hPIV3 CI, LS and LS-D556N HNs-NPs.
