# Supplementary material for: Unraveling dynamics of paramyxovirus-receptor interactions using nanoparticles displaying hemagglutinin-neuraminidase
Source: PLoS Pathog. 2024 Jul 25;20(7):e1012371. doi: 10.1371/journal.ppat.1012371 (PMC11302929; doi:10.1371/journal.ppat.1012371)
Supplement: S6 Fig — 7.43 x 10^8^ empty NPs (130 nm) were allowed to interact with 3’S(LN)3-, 6’S(LN)3-, or biotin-coated sensors. As a negative control, PBS was taken along. Nanoparticle numbers indicated here are according to NTA analysis, see also S1 Table. (DOCX) [file ppat.1012371.s006.docx]

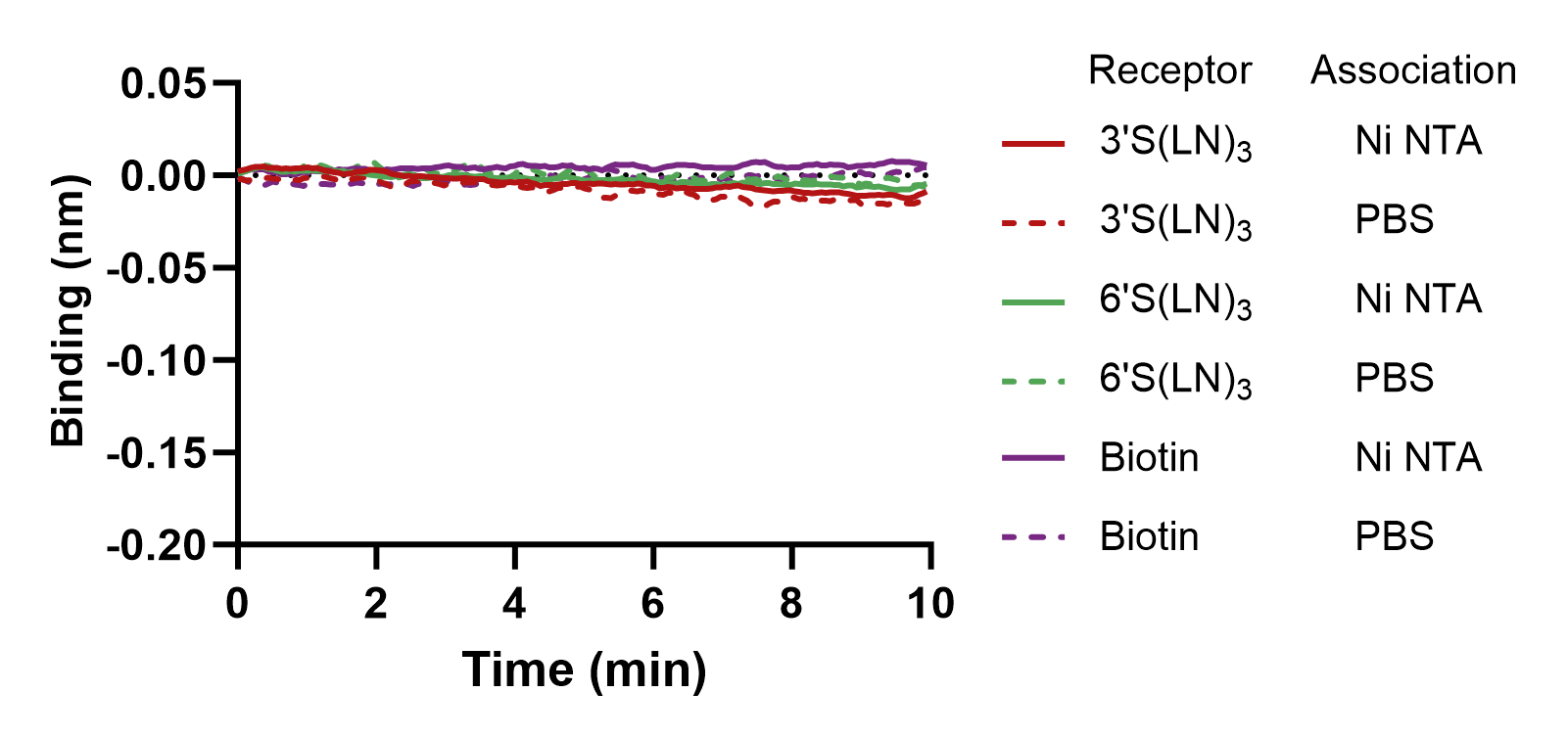


**S6 Fig. No binding of empty nanoparticles to the sensors.** 7.43 x 10^8^ empty NPs (130 nm) were allowed to interact with 3’S(LN)_3_-, 6’S(LN)_3_-, or biotin-coated sensors. As a negative control, PBS was taken along. Nanoparticle numbers indicated here are according to NTA analysis, see also S1 Table.
