# Supplementary material for: Unraveling dynamics of paramyxovirus-receptor interactions using nanoparticles displaying hemagglutinin-neuraminidase
Source: PLoS Pathog. 2024 Jul 25;20(7):e1012371. doi: 10.1371/journal.ppat.1012371 (PMC11302929; doi:10.1371/journal.ppat.1012371)
Supplement: S7 Fig — The sialidase activity of NDV HN-NPs (130nm, 7.43 x 10^8^ HN-NPs) or corresponding amount of soluble HNs (assuming 100% coupling efficiency) were determined by applying the 4-MUNANA fluorometric assay under standard conditions. Nanoparticle numbers indicated here are according to NTA analysis, see also S1 Table. (DOCX) [file ppat.1012371.s007.docx]

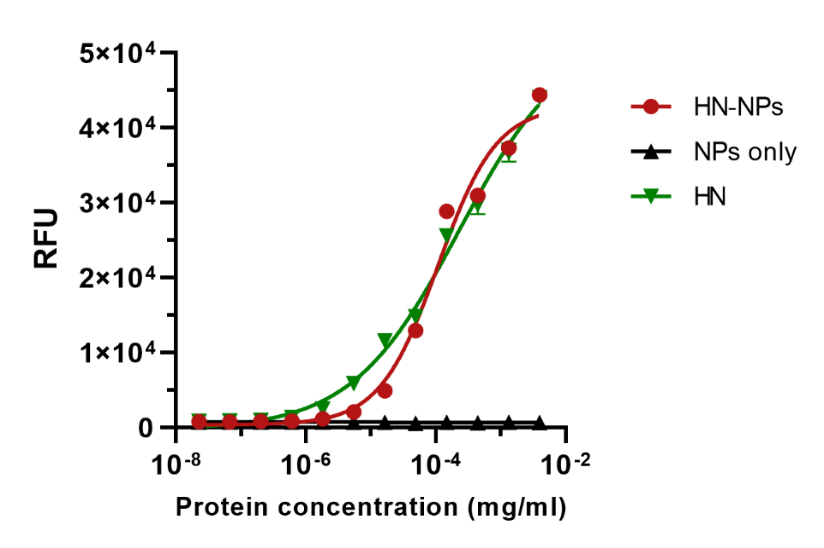


**S7 Fig. Sialidase activity of coupled and unconjugated HN.** The sialidase activity of NDV HN-NPs (130nm, 7.43 x 10^8^ HN-NPs) or corresponding amount of soluble HNs (assuming 100% coupling efficiency) were determined by applying the 4-MUNANA fluorometric assay under standard conditions. Nanoparticle numbers indicated here are according to NTA analysis, see also S1 Table.
