# Supplementary material for: Unraveling dynamics of paramyxovirus-receptor interactions using nanoparticles displaying hemagglutinin-neuraminidase
Source: PLoS Pathog. 2024 Jul 25;20(7):e1012371. doi: 10.1371/journal.ppat.1012371 (PMC11302929; doi:10.1371/journal.ppat.1012371)
Supplement: S8 Fig — Hemagglutination was performed starting with 7.43 x 10^8^ HN-NPs (130 nm, standard coupling) in 1st well or with the corresponding amount of soluble HNs (assuming a 100% coupling efficiency) using human erythrocytes. Nanoparticle numbers indicated here are according to NTA analysis, see also S1 Table. (DOCX) [file ppat.1012371.s008.docx]

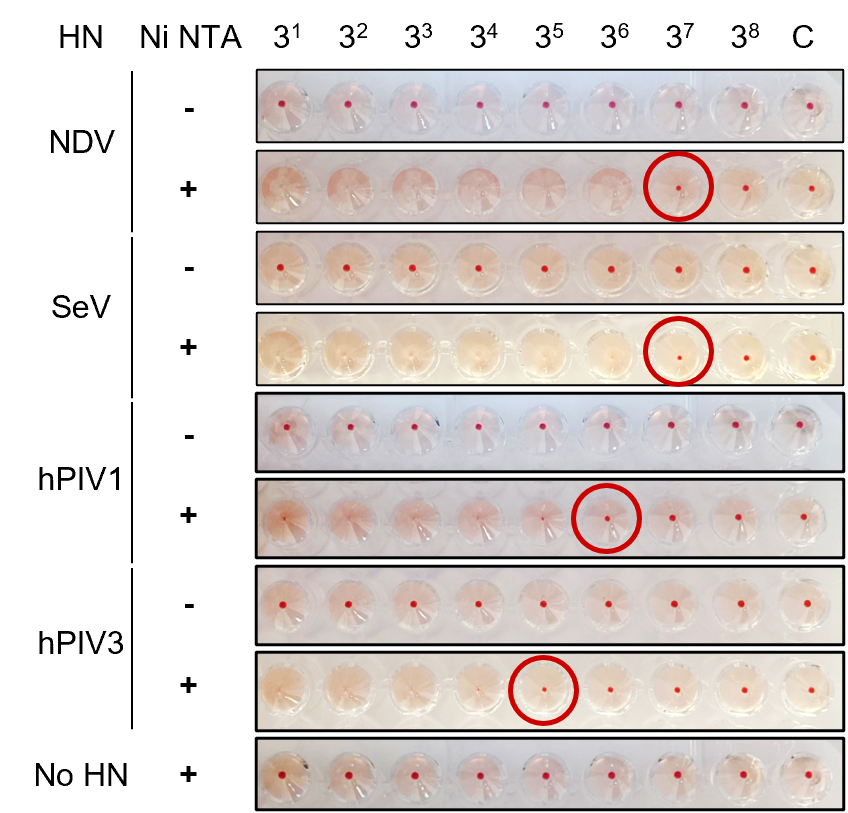


**S8 Fig. Hemagglutination analysis of the soluble HNs and HN-NPs.** Hemagglutination was performed starting with 7.43 x 10^8^ HN-NPs (130 nm, standard coupling) in 1st well or with the corresponding amount of soluble HNs (assuming a 100% coupling efficiency) using human erythrocytes. Nanoparticle numbers indicated here are according to NTA analysis, see also S1 Table.
