# Supplementary material for: Unraveling dynamics of paramyxovirus-receptor interactions using nanoparticles displaying hemagglutinin-neuraminidase
Source: PLoS Pathog. 2024 Jul 25;20(7):e1012371. doi: 10.1371/journal.ppat.1012371 (PMC11302929; doi:10.1371/journal.ppat.1012371)
Supplement: S9 Fig — Data were normalized to the max value of each row. The heat map was drawn using ChiPlot (https://www.chiplot.online/) (accessed on 09 January 2024). (DOCX) [file ppat.1012371.s009.docx]

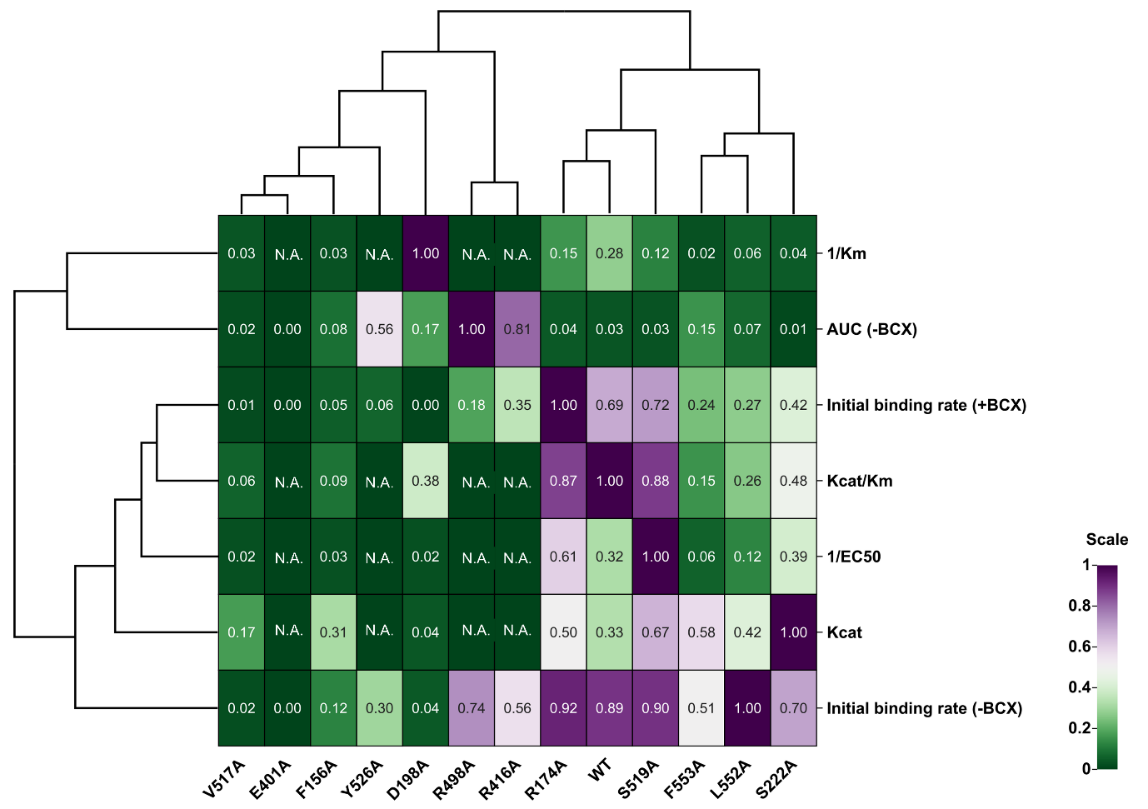


**S9 Fig. Correlation analysis of NDV HN WT and mutant proteins.** Data were normalized to the max value of each row. The heat map was drawn using ChiPlot (https://www.chiplot.online/) (accessed on 09 January 2024).
