# Supplementary material for: Unraveling dynamics of paramyxovirus-receptor interactions using nanoparticles displaying hemagglutinin-neuraminidase
Source: PLoS Pathog. 2024 Jul 25;20(7):e1012371. doi: 10.1371/journal.ppat.1012371 (PMC11302929; doi:10.1371/journal.ppat.1012371)
Supplement: S10 Fig — Differences between LS and CI sequences are highlighted in yellow, while the residues of the primary receptor binding site (site I) are highlighted in magenta. Residue 556 substituted in this study is indicated. hPIV3 LS (GenBank accession no. AET35008.1), hPIV3 CI GenBank accession no. AOO33557.1). (DOCX) [file ppat.1012371.s010.docx]

Supplementary Materials for


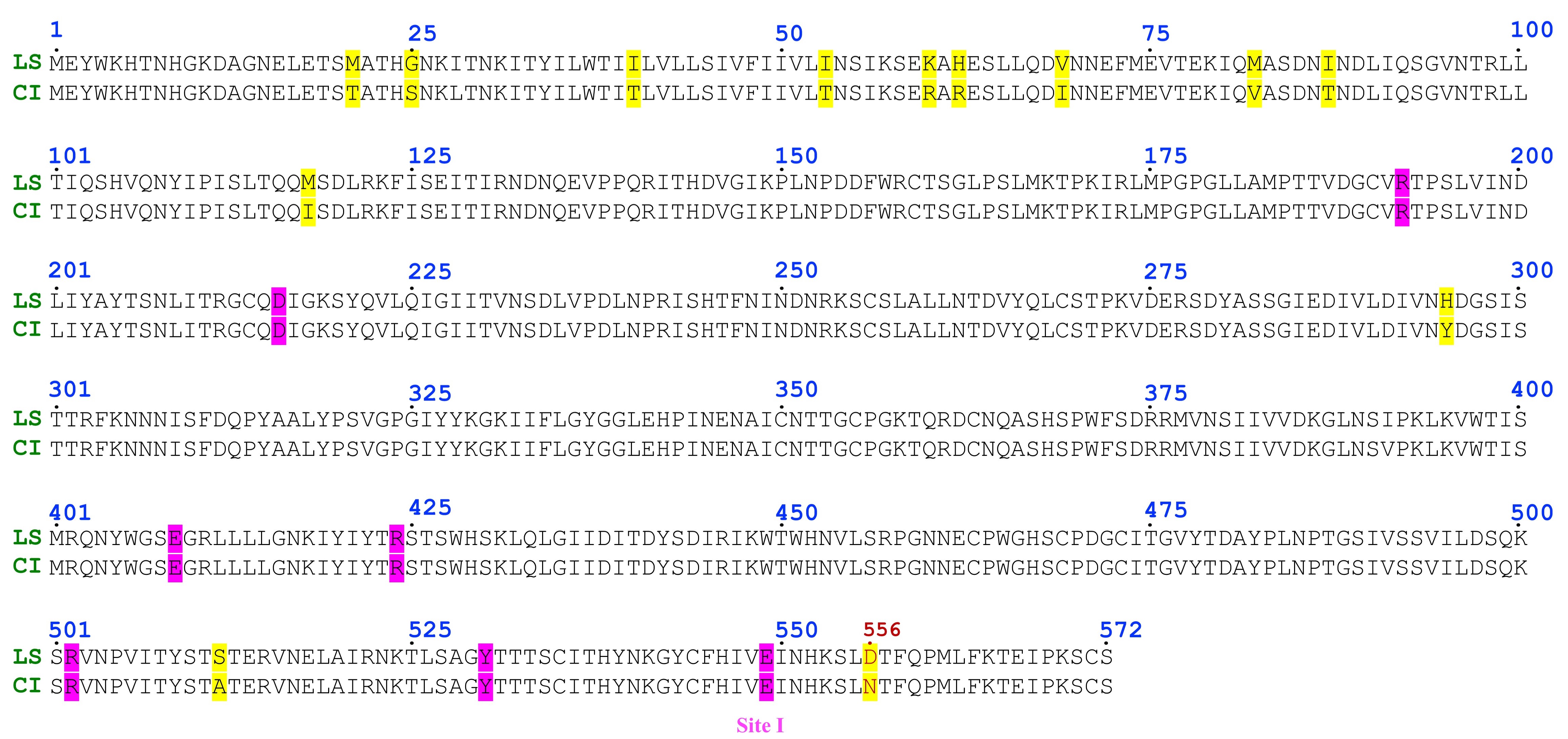


**S10 Fig. Sequence alignment of the HN proteins of the hPIV3 lab strain (LS) and clinical isolate (CI) used in this study.** Differences between LS and CI sequences are highlighted in yellow, while the residues of the primary receptor binding site (site I) are highlighted in magenta. Residue 556 substituted in this study is indicated. hPIV3 LS (GenBank accession no. AET35008.1), hPIV3 CI GenBank accession no. AOO33557.1).
