# Supplementary material for: Unraveling dynamics of paramyxovirus-receptor interactions using nanoparticles displaying hemagglutinin-neuraminidase
Source: PLoS Pathog. 2024 Jul 25;20(7):e1012371. doi: 10.1371/journal.ppat.1012371 (PMC11302929; doi:10.1371/journal.ppat.1012371)
Supplement: S11 Fig — Residues located in site I are colored in deepblue, and residues in site II are colored cyan. Residues linked with the same loop between site I and site II are shown in hotpink (PDB ID: 1USR). The thiosialoside is shown with yellow bonds. (DOCX) [file ppat.1012371.s011.docx]

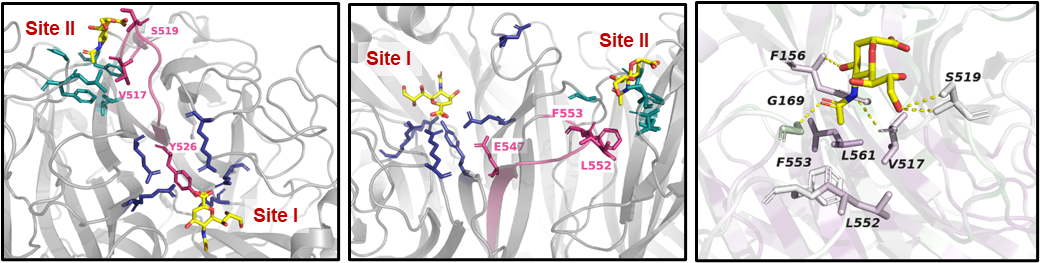


**S11 Fig.** Cartoon representation of the NDV HN Sia binding pockets. Residues located in site I are colored in deepblue, and residues in site II are colored cyan. Residues linked with the same loop between site I and site II are shown in hotpink (PDB ID: 1USR). The thiosialoside is shown with yellow bonds.
