# Supplementary material for: Unraveling dynamics of paramyxovirus-receptor interactions using nanoparticles displaying hemagglutinin-neuraminidase
Source: PLoS Pathog. 2024 Jul 25;20(7):e1012371. doi: 10.1371/journal.ppat.1012371 (PMC11302929; doi:10.1371/journal.ppat.1012371)
Supplement: S1 Table — (DOCX) [file ppat.1012371.s012.docx]

**S1 Table. Nanoparticle concentration and HN tetramers density on single particles.**

| Ni-NTA Type | Diameter (nm) | **Concentration (particles/ml)** | | Particles used for coupling 0.45 µg HN (manufacturer’s specifications) | Particles used for coupling 0.45 µg HN (based on NTA) | **Assuming 100% coupling** | |
| --- | --- | --- | --- | --- | --- | --- | --- |
|  |  | manufacturer’s specifications | NTA |  |  | HN tetramers/particle (manufacturer’s specifications) | HN tetramers/particle (based on NTA) |
| Gold | 100 | 1.92E+11 | 5.88E+10 | 1.44E+09 | 4.41E+08 | 6.74E+02 | 2.20E+03 |
|  | 80 | 3.91E+11 | 5.62E+10 | 1.44E+09 | 2.07E+08 | 6.74E+02 | 4.69E+03 |
|  | 60 | 9.80E+11 | 9.51E+10 | 1.44E+09 | 1.40E+08 | 6.74E+02 | 6.94E+03 |
|  | 40 | 3.58E+12 | 2.10E+11 | 1.44E+09 | 8.45E+07 | 6.74E+02 | 1.15E+04 |
|  | 30 | 8.96E+12 | 2.09E+11 | 1.44E+09 | 3.36E+07 | 6.74E+02 | 2.89E+04 |
| Normal | 130 | 3.50E+12 | 9.90E+10 | 2.63E+10 | 7.43E+08 | 3.69E+01 | 1.31E+03 |
|  | 250 | 4.90E+11 | 9.42E+10 | 2.63E+10 | 5.05E+09 | 3.69E+01 | 1.92E+02 |
