# Supplementary material for: Unraveling dynamics of paramyxovirus-receptor interactions using nanoparticles displaying hemagglutinin-neuraminidase
Source: PLoS Pathog. 2024 Jul 25;20(7):e1012371. doi: 10.1371/journal.ppat.1012371 (PMC11302929; doi:10.1371/journal.ppat.1012371)
Supplement: S2 Table — (DOCX) [file ppat.1012371.s013.docx]

**S2 Table. Summary of mutagenesis studies performed for site I and site II/interface of NDV HN**
