# Supplementary material for: Unraveling dynamics of paramyxovirus-receptor interactions using nanoparticles displaying hemagglutinin-neuraminidase
Source: PLoS Pathog. 2024 Jul 25;20(7):e1012371. doi: 10.1371/journal.ppat.1012371 (PMC11302929; doi:10.1371/journal.ppat.1012371)
Supplement: S3 Table — (DOCX) [file ppat.1012371.s014.docx]

**S3 Table. Statistical analysis of initial binding rates of NDV HN-NPs in the absence or presence of BCX2798 (based on results shown in Figs 5 and 6)**

ns, P>0.05; *, P≤0.05; **, P≤0.01; ***, P≤0.001; ****, P < 0.0001.
